# The effectiveness of vitamin D supplementation in patients with end-stage knee osteoarthritis: Study protocol for a double-blinded, randomized controlled trial

**DOI:** 10.1371/journal.pone.0309610

**Published:** 2024-10-21

**Authors:** Qian-Wen Wang, Michael Tim-Yun Ong, Gene Chi-Wai Man, Yi-Man Yeung, Xin He, Ben Chi-Yin Choi, Jonathan Patrick Ng, Daniel Kam-Wah Mok, Tsz-Ping Lam, Patrick Shu-Hang Yung

**Affiliations:** 1 Department of Orthopaedics and Traumatology, Faculty of Medicine, The Chinese University of Hong Kong, Prince of Wales Hospital, Hong Kong SAR, China; 2 Department of Orthopaedics and Traumatology, Prince of Wales Hospital, Hong Kong SAR, China; 3 Department of Food Science and Nutrition, the Hong Kong Polytechnic University, Hong Kong SAR, China; University of Naples Federico II: Universita degli Studi di Napoli Federico II, ITALY

## Abstract

Osteoarthritis (OA) knee is one of the most common chronic degenerative conditions that imposes clinical and economic burdens on individuals and societies worldwide. Previous studies showed vitamin D levels correlated positively with lean muscle mass and grip strength, implying that vitamin D supplementation may improve muscle health in knee OA subjects. This randomized controlled trial (RCT) aims to compare the effects of vitamin D supplementation on knee muscle strength, physical function, pain, and sarcopenia status in patients with end-stage knee OA. Patients and outcome assessors will be blinded to group allocation. Fifty-six end-stage knee OA patients with vitamin D insufficiency fulfilling our inclusion criteria will be invited to participate in this study. Patients will be randomly assigned to take vitamin D supplementation (4,000 IU capsule daily) or placebo for six months. Measurements will be taken at baseline, three and six-month after the commencement of the vitamin D supplement, and 6-month after the interventional period. The primary outcome includes the isometric quadriceps and hamstring muscle strength measured by a hand-held dynamometer. Secondary outcomes include pain, performance-based and self-reported physical function and sarcopenia status. The success of this study will provide scientific evidence of whether the relatively cheap and well-tolerated vitamin D supplement can improve quadriceps muscle strength, physical function, pain symptoms, and sarcopenia status of this increasingly large population for end-stage knee OA patients. The study has great clinical significance given Hong Kong’s lengthy and growing waiting list for complete knee replacement procedures.

**Trial registration:** The trial was registered on clinicaltrials.gov (NCT05981534) on 31^st^ July 2023.

## Introduction

One of the most prevalent chronic degenerative disorders is osteoarthritis (OA) of the knee. Older adults who experience it become disabled, causing discomfort and stiffness. Among persons over 60, the prevalence of radiologic knee OA was 37.4%, while that of symptomatic knee OA was 12.1% [1]. With just 4300 total knee replacements conducted in 2021, the Hospital Authority (HA) had over 26,000 patients on its waiting list for knee total knee replacements (TKRs), meaning that the estimated waiting period in Hong Kong was about 89 months [2]. The number of people with end-stage knee OA who are waiting for surgery is anticipated to rise sharply as the population ages. While waiting for TKR, patients with end-stage OA can benefit from additional therapies to help them function better and maintain overall health.

Patients with end-stage knee OA frequently give up on physical activity to prevent joint stiffness and pain, which can negatively impact overall muscle health and localized knee function. As people age and become less active, their muscular mass and strength gradually decrease to an unusually low level known as "sarcopenia" [3]. Older adults with sarcopenia are more likely to have a decline in functional outcomes and a greater death rate, which puts a strain on their finances and healthcare system [4]. On the other hand, sarcopenia was found in 32.8% of patients with end-stage knee OA in a previous study on the prevalence of sarcopenia in end-stage knee OA patients. These patients likewise recovered more slowly following TKR [5]. A recent study categorized “sarcopenic knee OA” patients as a new subgroup with a higher risk of falling and associated risk factors than patients with sarcopenia or knee OA alone [6]. Strengthening exercise has been indicated to improve muscle mass, strength, and gait speed among sarcopenic patients [7]. However, the individual responses to exercise are variable [8].

The common therapies for knee OA include non-pharmacologic, pharmacologic, surgical, and intra-articular routes or the combined treatment to reach the optimal outcomes [9]. The initial treatment line for all OA stages is always non-pharmacologic and includes patient education, self-management, exercise and physical activity [10]. Pharmacologic interventions, such as NSAIDs, are required when OA worsens, and symptoms such as pain and stiffness become more severe [11]. For end-stage knee OA patients, total knee replacement surgery is one of the most effective surgical interventions for pain relief and functional recovery [12]. Other therapies, such as functional biomaterials and injectable hydrogel microspheres, that target improving the self-repair ability of articular cartilage have recently shown great therapeutic potential in the animal testing stage [13, 14]. Applying microenvironment-responsive nanosystems in OA treatment also has significant promise as a forward-thinking strategy for creating sophisticated therapeutic interventions, emphasising their potential to provide targeted and controlled drug delivery [15]. It offers valuable information on the development of nanosystems for improved treatment outcomes.

Although the benefits of vitamin D on musculoskeletal health have long been known, its impact on muscular function is also receiving more and more attention. By acting on certain vitamin D receptors in myocytes, vitamin D directly influences muscle growth. It has been observed that individuals with adequate vitamin D levels have larger, more numerous, and stronger muscular fibers. Additionally, vitamin D has positive benefits through its interaction with myokines such irisin and myostatin. Biological, clinical, and epidemiological data indicate the link between vitamin D and an elevated risk of sarcopenia in the elderly. It has been demonstrated that elderly patients with vitamin D deficiency are prone to sarcopenia [16]. In addition to having a negative effect on cartilage thickness, low blood vitamin D has been linked in a research to accelerated radiographic knee OA development [17]. Up to 62% of older adults with OA were found to have vitamin D insufficiency [18], and the risk of low vitamin D is exceptionally high compared to the average population, as many of these patients are confined indoors due to the decreased mobility limited by pain [19]. Thirty-six percent of Chinese adults over 65 who were evaluated had vitamin D deficiency (25(OH)D <20 ng/mL) [20]. According to the findings from previous systematic review, vitamin D supplementation can help people with knee OA with their pain and function [21]. In addition, those with end-stage knee OA would experience increased discomfort, which could result in reduced mobility, an increased risk of sarcopenia, and a vitamin D deficiency that needs to be addressed.

### Objectives

The study aims to investigate the effect of vitamin D supplementation on knee muscle strength, physical function, pain symptoms, and sarcopenia status in end-stage OA knee patients.

## Materials and methods

### Criteria, study timeline, ethics approval, consent form, clinical registration

**Fig 1:**
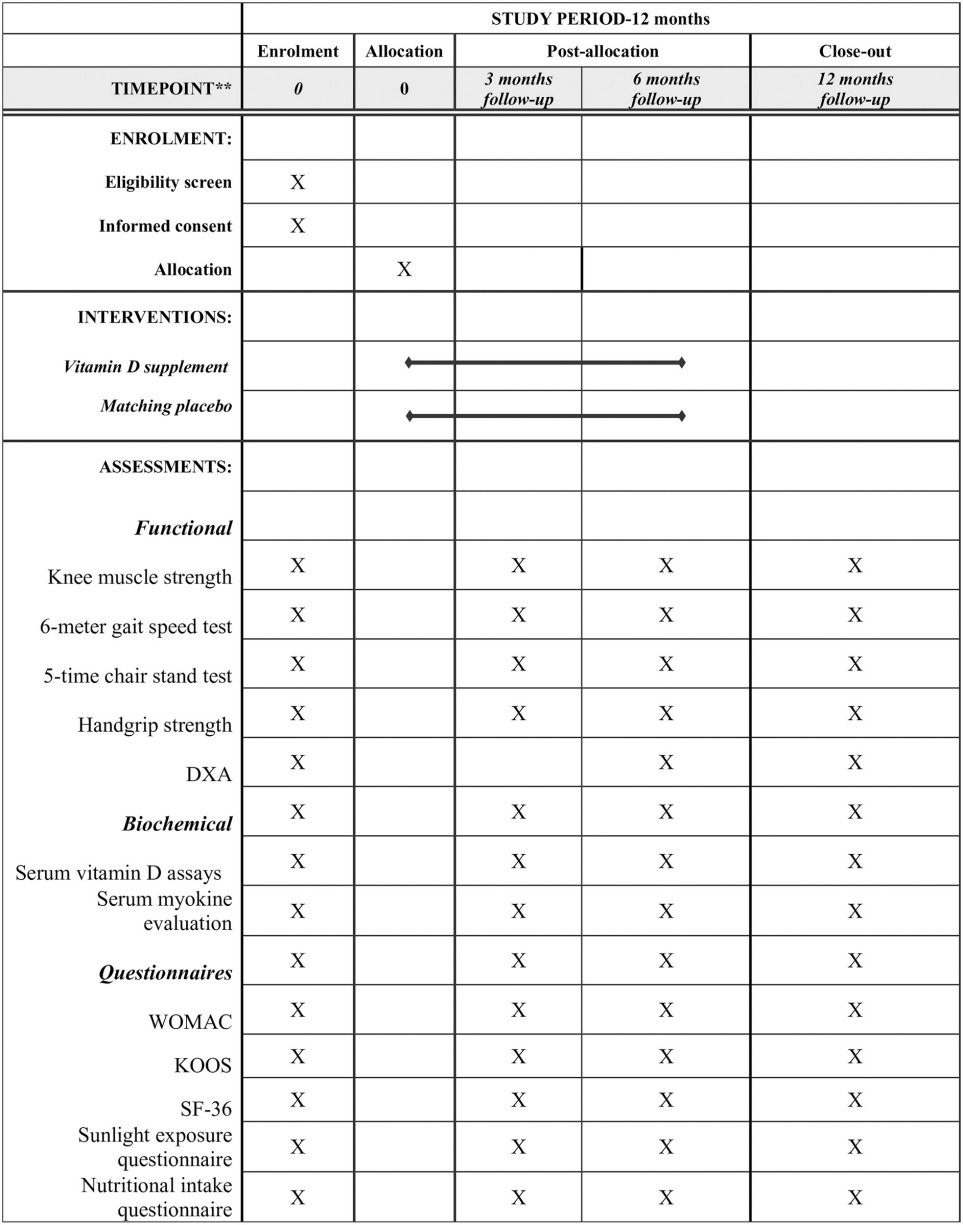
SPIRIT schedule of enrolment, intervention, and assessments. This figure includes participants’ recruitment, allocation, intervention, and outcome assessments.

The Standard Protocol Items: Recommendations for Interventional Trials (SPIRIT) criteria are adhered to by this protocol (see the SPIRIT checklist in S1 File) [22]. Fig 1 shows the SPIRIT schedule of enrolment, intervention, and assessments.

Initial ethics approval and consent to participate have been obtained from the Joint Chinese University of Hong Kong–New Territories East Cluster Clinical Research Ethics Committee (CREC no. 2022.252), in accordance with the declaration of Helsinki (See S2–S4 Files with initial ethic approval, the updated ethics approval, Chinese and English versions of consent forms, and approved protocol). The trial was registered on clinicaltrials.gov (NCT05981534).

All participants will be required to provide written informed consent prior to enrollment. The informed consent process will be conducted by a trained research staff member, who will explain the study purpose, procedures, potential risks and benefits, and the participants’ right to withdraw from the study at any time without penalty. Participants will be given sufficient time to review the informed consent form and ask any questions they may have before signing.

#### Potential ethical concerns

*Risks of vitamin D supplementation*. While vitamin D supplementation is generally considered safe, there is a small risk of hypercalcemia or other adverse effects. Participants will be closely monitored. Any participants who develop significant adverse events will be withdrawn from the study and provided with appropriate medical care.

*The burden on participants*. The study involves multiple visits and assessments over 12 months, which may be burdensome for some participants. To mitigate this, participants will have flexible scheduling to accommodate their needs.

*Confidentiality and data protection*. All participant data will be de-identified and stored securely. Only the research team will have access to the identifiable data, and any publications or presentations will ensure participant anonymity.

### Trial design

The study is a randomized, double-blinded, controlled study that aims to compare the effects of vitamin D supplementation on knee muscle strength, physical function, pain, and sarcopenia status in patients with end-stage knee OA at 3 months and 6 months after the commencement of vitamin D supplement, and 6-month after the interventional period.

### Participants and setting

Based on our inclusion criteria, participants will be recruited with consent from the outpatient clinic of the Department of Orthopaedics and Traumatology at the Prince of Wales Hospital (affiliated with the Chinese University of Hong Kong).

### Eligibility criteria

To be enrolled in this trial, the following eligibility criteria, assessed at screening, will be met:

#### Inclusion criteria

The inclusion criteria are as follows:

1. Male and female patients aged over 50 and clinically diagnosed with knee OA with Kellgren-Lawrence (KL)grade 3 or above at least one side.
2. Patients are on the waiting list for TKR at Prince of Wales Hospital
3. Walk unaided for 6 meters
4. Able to comply with the assessments and has given oral and written consent
5. Patients with vitamin D insufficiency and deficiency at the baseline measurement (25(OH)D <30 ng/mL)

#### Exclusion criteria

The exclusion criteria are as follows:

1. Patients with connective tissue disorders or myositis condition
2. History of any Hip & Knee surgery
3. Patients with malnutrition were assessed by Mini-Nutritional assessment.
4. Patients with acute immobility (i.e., post-hip fracture or post-acute hospital admission)
5. Patient scheduled for TKR within six months
6. Patients already taking vitamin D supplements
7. Patients with a known contraindication to vitamin D treatment (such as allergy)
8. Patients who have renal impairment with glomerular filtration rate < 30 ml/minute

### Recruitment

Based on the inclusion and exclusion criteria, qualified patients will be recruited from the outpatient clinic with written permission from the Prince of Wales Hospital’s Department of Orthopaedics and Traumatology. Additionally, the center’s employees will help find qualified patients and refer them to us for screening. Then, they will then be randomly using a computer randomization program to allocate the participants as either to receive the vitamin D supplement or placebo The following basic patient demographics will be noted: age, gender, ethnicity, occupation, BMI, and alcohol and tobacco use. The Clinical Management System, Hospital Authority, the main computerized database for Hong Kong’s public hospitals, will also be used to verify and record medical histories.

### Informed consent

Before participants takes part in the study, trained research assistants will get their written and oral informed permission. First, eligible participants will receive a thorough explanation of our study from our research assistants. All patients must give their informed consent in order to attend the trial. The Declaration of Helsinki will be followed in the conduct of the study. The ethics committee has granted ethical approval.

Informed consent also covers agreement for biological specimens and participant data. Each participant will be assigned an ID number, and private information like name, address, and phone number will be kept private before, during, and after the research.

### Randomization and allocation concealment

A total of 56 patients will be enrolled. Participants will be randomized into 1:1 allocation, blocked randomization with 28 participants in the Vitamin D group and 28 participants in the control group. An independent research staff will assign participants to interventions.

The randomization will be done using a computer randomization program by an independent statistician before the intervention. To ensure blindness, a randomization code will be assigned to every participant. Only once the database has been locked will the randomization code be broken. Assessment and data analysis are conducted by different research staffs blinded to the patient’s randomization assignment.

### Blinding

The participants’ group assignment will remain a secret to all parties involved—the researchers, statisticians, research assistants, and patients—during the study and assessment. The research assistant who assists in obtaining consent oversees the study’s advancement and reports any adverse events will not be involved in the data analysis. The assessor, a distinct knowledgeable research assistant, will not be blind to the randomization status and will not participate in the intervention. The statistician responsible for the randomization process is not involved in the data analysis or other project-related tasks.

### Procedure for unbinding if needed

Unless a major unfavorable event happens, such as hypercalcemia or kidney stones, blindness will be kept in place under normal conditions. The trial’s unblinded participants will thereafter be terminated, and their medical issues will be treated appropriately.

### Study interventions

Pirotta and his colleagues found that daily supplementation with 2,000 IU vitamin D3 for 10 weeks in older adults significantly increased knee extension muscle strength by 8–11% but did not reach the statistically significant group difference compared with the placebo group [23]. In another previous study, 50 older adults with vitamin D levels < 20 ng/ml took 4000IU daily vitamin D supplement, and 78% of aged older adults had 25(OH)D levels ≥ 30 ng/mL after 6 months [24]. The doses of 4000 IU vitamin D3 daily have also been reported to be required to achieve plasma 25(OH)D levels associated with lowest disease risk in observational studies in older adults [25]. Vitamin D group will receive 4000 IU/day for six months, modified from the earlier research [23–25]. Subjects will be randomized into 2 study groups: (1) Intervention group: patients receive daily dose of 4000 IU of vitamin D3 supplements. (2) Control group: patients receive placebo. Supplements will be dispensed to participants in baseline and 1^st^ follow-up to increase the subject compliance.

**Fig 2:**
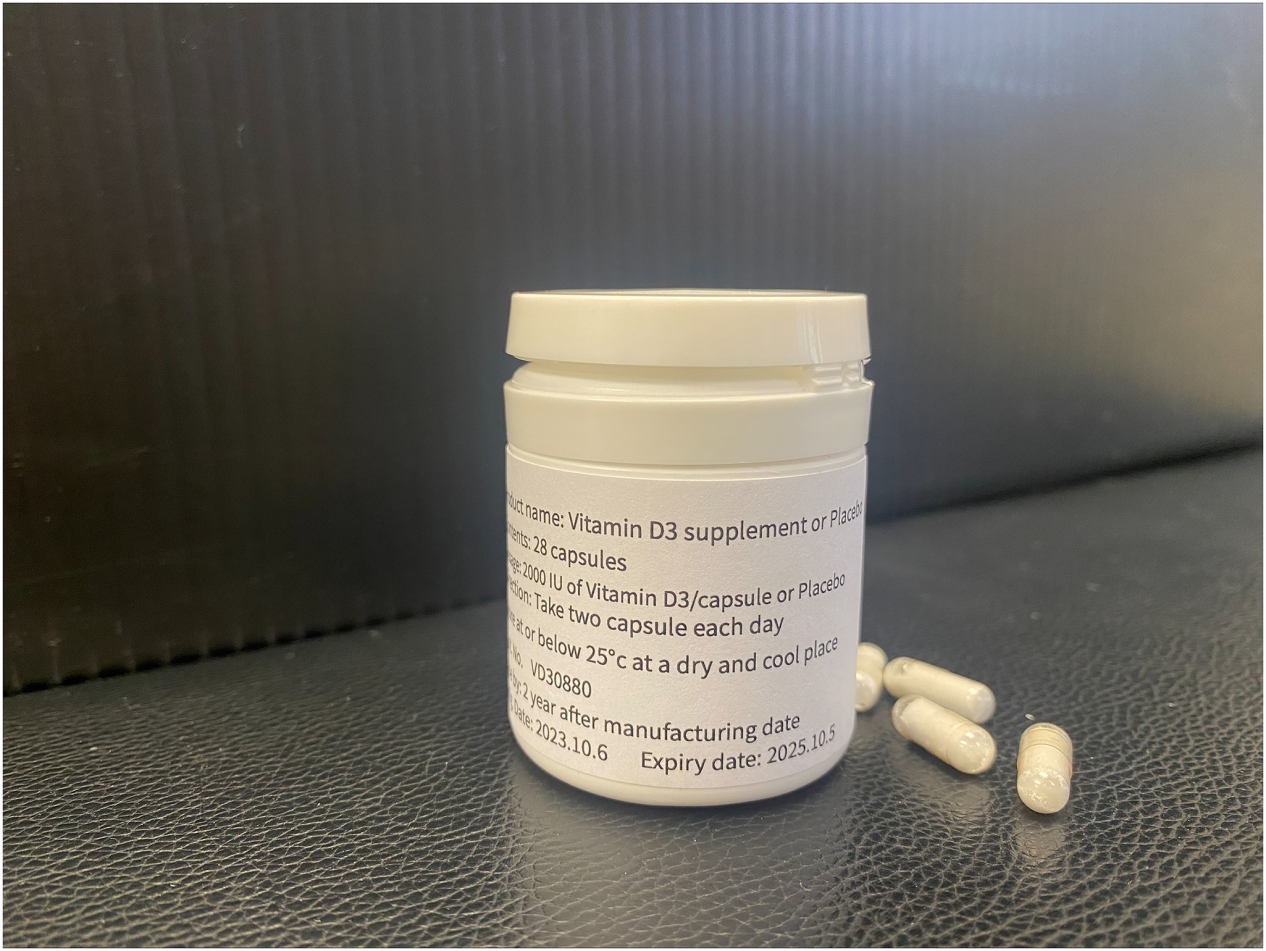
Vitamin D3 supplement or placebo.

All study capsules, including the placebo, will be manufactured according to Good Manufacturing Practice (GMP) guidelines for quality assurance (see Fig 2). All capsules purchased from SODX Co., Ltd, Osaka, Japan, and patients in the control arm will receive an identical inert placebo provided by the same company. As a placebo will look and taste like vitamin D3, this can ensure the participants are blinded to the treatment. Utilizing a placebo that closely mimics the appearance and flavor of vitamin D3 will effectively blind the participants to their treatment.

### Adverse events, safety and modifications

The upper tolerable intake level for vitamin D in the general population has been set at 4000/IU daily [26]; as this is a long-term treatment program, the self-reported harm effects remain uncertain. Research staff will monitor and handle participant concerns regarding discomfort. Medical staff will also evaluate whether immediate removal from the study is necessary for their best interest to safeguard their health. Any unfavorable incidents or adverse events that develop throughout the study will be brought to the attic committee.

A significant adverse event must be notified to the Hong Kong Hospital Authority Research Ethics Committee within twenty-four hours of the incident. Until the serious adverse event is resolved or finished, the primary investigator will be in charge of managing it. The trial steering group and investigators will determine whether a minor or significant adverse event is related to the study intervention. The principal investigator will promptly stop the intervention if the reported event is connected to it and advise the patient to seek medical attention.

### Adherence and satisfaction

Patients will be required to keep personal diaries with information on their medications. Additionally, the study team will increase adherence by calling the patients once a week to check on them. The research staff will contact patients who miss a scheduled session and ask them to reschedule at a different time within a week. When a patient refuses to participate in the study, the researchers will only withdraw them from it after making several unsuccessful attempts to get in touch with and persuade them. Participants can also stop participating in the study at any time for any reason; if they do, they will be asked if they want to be contacted again according to the trial schedule. Participants will be encouraged to report the incidence of falls in the study period.

Satisfaction and feedback will be recorded based on the following questions at 0-month, 3-month, 6-month and 12-month follow-up assessments.

1. Are you satisfied with the vitamin D supplementation? (1 = Very Dissatisfied, 2 = Dissatisfied, 3 = Neutral, 4 = Satisfied, 5 = Very Satisfied)
2. How effective do you feel the vitamin D supplementation has managed your knee osteoarthritis symptoms? (1 = Not effective at all, 2 = Slightly effective, 3 = Moderately effective, 4 = Highly effective, 5 = Extremely effective)
3. Do you have any additional comments or feedback regarding your experience with the vitamin D supplementation?

### Relevant concomitant care permitted or prohibited during the trial

Participants could maintain their regular course of care and medication schedule except for other Vitamin D supplements during the study, and physicians are advised to manage participants in accordance with standard practice while taking the previously mentioned precautions into account.

### Provisions for post‑trial care

Not applicable, since ancillary and post-trial care is provided within the standard care.

### Outcomes and outcome assessments

The primary outcomes will be muscle strength using quadriceps and hamstring muscle assessments, and the secondary outcomes will be the post-intervention change in muscle strength function from baseline using 5-time chair stand test, sarcopenia assessment by Dual Energy X-ray Absorptiometry (DXA), handgrip strength and 6-meter gait speed test, six questionnaires: Western Ontario and McMaster Universities Osteoarthritis Index (WOMAC), Knee Injury and Osteoarthritis Outcome Score (KOOS), Short-Form 36 (SF-36), International Physical Activity Questionnaire (IPAQ), Nutritional Intake and Sunlight Exposure questionnaire (Questionnaires can be seen in S5 File), and biochemical assays including serum Vitamin D level and myokines. Blood samples will be taken under non-fasting conditions. The serum obtained will be immediately stored at -80°C until analysis.

Outcome assessments of the patients will be performed at baseline (0 month), 3 months and 6months after the commencement of vitamin D supplement, and 6-month after the interventional period.

#### Knee muscle strength

**Fig 3:**
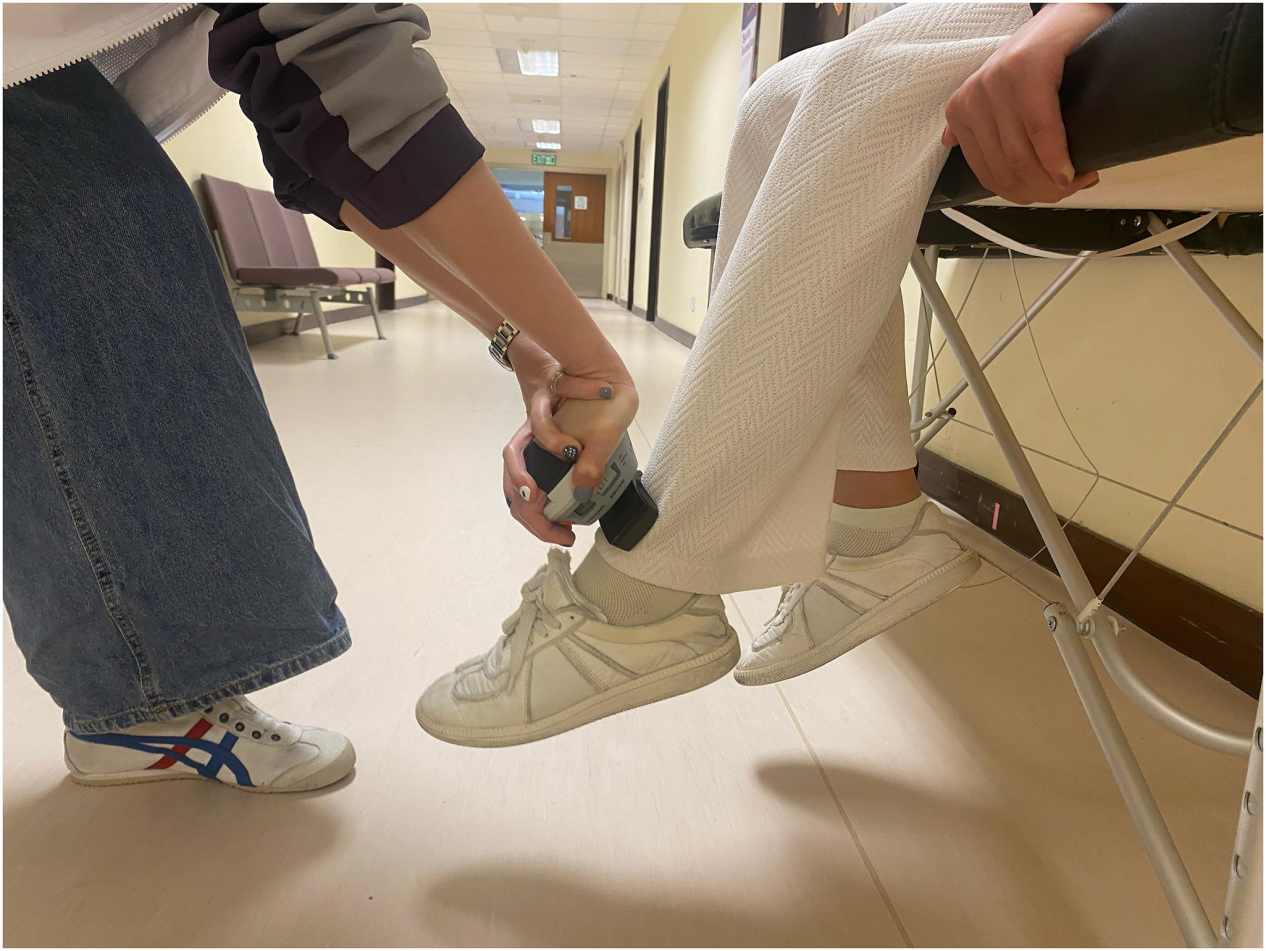
Quadriceps muscle strength test.

**Fig 4:**
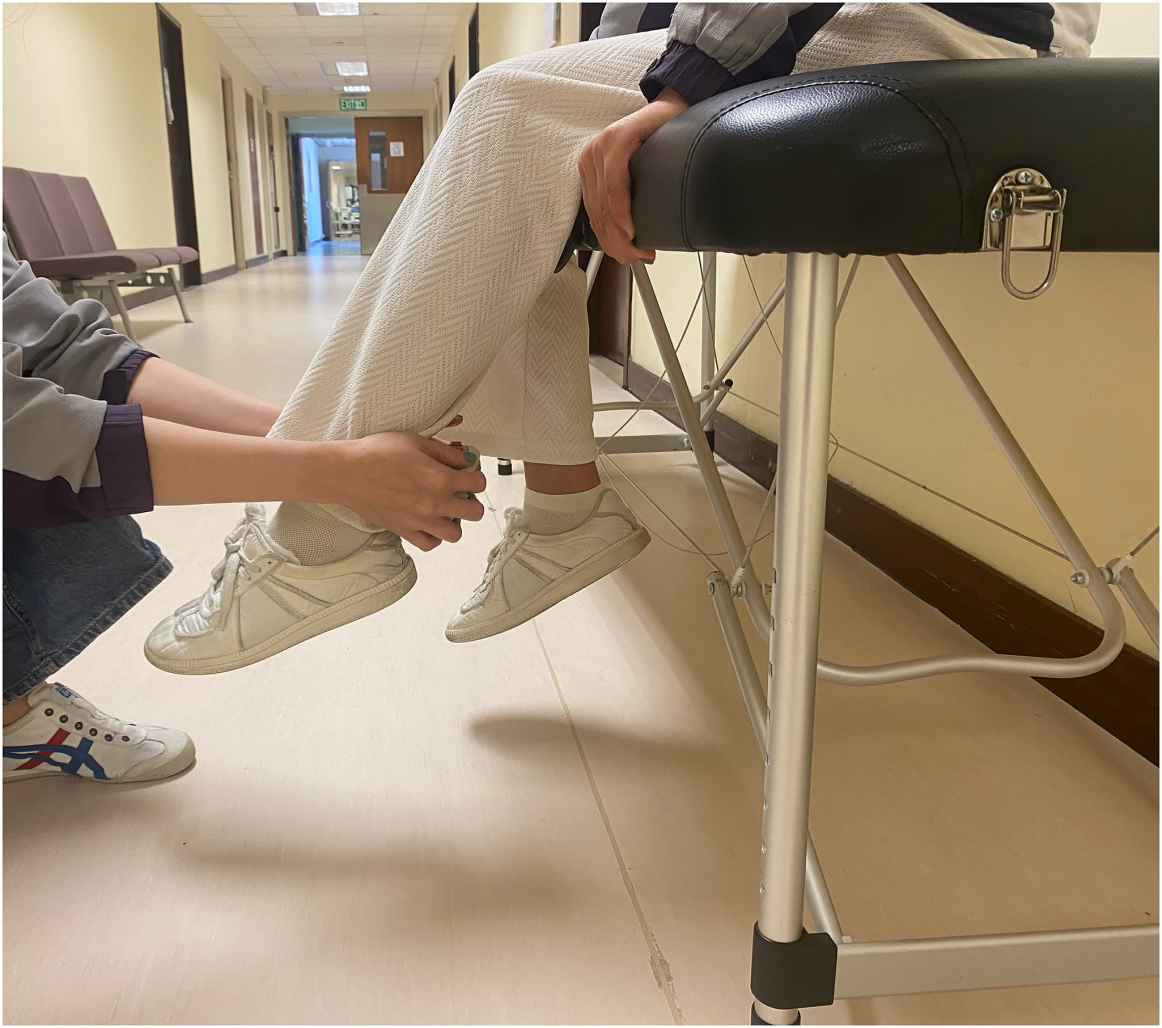
Hamstring muscle strength test.

The primary outcome will be obtained by measurement of knee muscle strength using a hand-held dynamometer (Hoggan Scientific, Salt Lake City, UT, USA) to assess maximal voluntary quadriceps and hamstring muscle strength. Each participant will be placed on an examination table with their feet elevated and their knees bent at a 60°. The maximal voluntary quadriceps muscle strength on the anterior aspect of the distal tibia (see Fig 3) and the maximal voluntary isometric hamstring muscle strength will be measured using the hand-held dynamometer placed on the posterior portion of the lower leg (see Fig 4). For stability, participants will hold onto the examination table with their hands. They will then be told to flex or extend their knee "as hard as possible" into the hand-held dynamometer. For five seconds, participants will keep applying force to the hand-held dynamometer, and the maximum force they can muster during the experiment will be noted. All tests will involve maximal voluntary isometric contractions. Both limbs will be assessed to record. Two testing trials will be completed. The maximum knee extensor and flexor strength were calculated to obtain an overall muscle strength value (kgf) divided by the patient’s body mass.

#### 6-meter gait speed test

A reliable method for determining gait speed is the 6-meter timed walking test. However, because of space constraints and the comprehensiveness of the exam for OA patients, the 6-meter test has been shown to be a viable and trustworthy alternative [27]. The successful two trials will be measured, and the minimum time needed will be recorded.

#### 5-time chair stand test

This test is a reliable and validated tool for the measurement of lower limb strength [28]. Patients will sit on a chair with their feet shoulder-width apart and their arms resting on their shoulders. They will complete five reputations across two trials and one or two repeats of the test to get comfortable with it. We’ll keep track of time in seconds.

#### Dual energy X-ray absorptiometry (DXA)

For a single, 20-minute session of muscle mass evaluation, the radiation dose is less than 25μSv, falling within the acceptable limit. Two copies of the patient’s body composition including total fat percentage, body mass index, and—most importantly for this study—lean appendicular muscle mass will be printed following each DXA scan.

#### Handgrip strength

The maximal handgrip strength (kg) will be evaluated for knee OA patients using the handgrip dynamometer. After three iterations of the test, the dominant hand’s average will be determined.

#### Definition of sarcopenia

Sarcopenia was defined by following the Asian Working Group for Sarcopenia (AWGS2) algorithm [29]. A patient with low muscle mass, low muscle strength, and/or low physical performance was categorized as having sarcopenia. Low muscle mass was defined as height-adjusted muscle mass by DXA < 7.0 kg/m^2^ for men and < 5.4 kg/m^2^ for women; low muscle strength was defined as grip strength < 28 kg for men and < 18 kg for women; and low physical performance as gait speed < 1.0 m/s for both men and women.

#### Western ontario and mcmaster universities osteoarthritis index (WOMAC)

Self-reported pain, stiffness, and physical function will be measured using WOMAC, which has a total score of 96. A higher score denotes an individual with greater disability [30].

#### Knee injury and osteoarthritis outcome score (KOOS)

Pain, other symptoms, function in daily living (ADL), function in sport and recreation (Sport/Rec), and knee-related quality of life (QOL) are the five separately subscales used in KOOS, which was designed to assess symptoms and function in patients with knee injury and osteoarthritis. Lower scores mean worse condition of the patients [31].

#### Short-form 36 (SF-36)

The validated and dependable Short-Form 36 questionnaires will be used to measure health-related quality of life [30]. The questionnaires consist of 36 items that measure a patient’s functional health and well-being from the perspective of the patient. The patients’ physical and mental health are summarized by this trustworthy and established assessment.

#### International physical activity questionnaire (IPAQ)

We will also evaluate the level of physical activity and inactivity for each patient. Three categories—Category 1 Inactivity, Category 2 Minimally Active, and Category 3 HEPA Active—will be calculated by this questionnaire based on the weekly activity report from patients [32].

#### Nutritional intake questionnaire

The last twelve months’ retrospective means of calculation will be the basis for evaluating habitual dietary consumption. We will employ a Food Frequency Questionnaire (FFQ) that was previously validated using information from the Hong Kong Adult Dietary Survey [33].

#### Sunlight exposure questionnaire

This study will employ the Chinese version of the Sunshine Exposure Questionnaire, which is a reliable tool for measuring lifetime solar exposure among Chinese women in Hong Kong [34].

#### Serum myokine evaluation

Blood taking (5 ml) will be performed. After centrifugation, the serum will be stored in a -80º freezer until needed. The Human Myokine Magnetic Bead Panel (Millipore) with Bioplex-200 bead-based suspension assay system (LKSIHS core facilities) or enzyme-linked immunosorbent assay (ELISA) will be used for the quantitative investigation of myokines and proteins relevant to muscle metabolism. These include Brain-derived neurotrophic factor (BDNF), Fibroblast growth factor-21 (FGF-21), Interleukin-6 (IL-6), IL-15, Irisin, Insulin-like growth factor 1 (IGF-1), FGF-2, IL-8, Follistatin, Musclin, Myonectin, Decorin, Meteorinlike, Osteopontin, Secreted protein acidic and rich in cysteine (SPARC), Klotho, Procollagen type III N-terminal peptide (P3NP), and C-terminal of troponin T1 (TNNT1).

#### Serum vitamin D assays

A commercial 25(OH) Vitamin D ELISA kit (Abcam ab213966) will be used to assess the serum 25(OH)Vit-D levels in accordance with the manufacturer’s instructions. This will yield a quantitative result of 25(OH) Vitamin D3 and 25(OH) Vitamin D2. 1.98 ng/ml of sensitivity (range: 0.5 ng/ml to 1010 ng/ml). When baseline serum 25(OH)D levels are less than 30 ng/mL, it is considered as vitamin D insufficiency [35].

### Data management and confidentiality

To guarantee the accuracy of outcome evaluations and data collection, a research assistant will get training. Any problems affecting the quality of research will be monitored by the ethics committee, and appropriate action will be taken if required. Patients may drop up the research at any moment, for any reason, and it won’t have an impact on their legal or medical rights. An identity code will be given to every patient. The database and list of patient identifying codes will be protected.

### Data access

The cleansed data sets will be made available to all Principal Investigators. Each and every data set will have a password. Data distributed to project team members will be blinded of any participant identifying information in order to maintain confidentiality.

### Data statement

The results will be presented at international scientific conferences and through publications in peer-reviewed journals.

### Plans for collection, laboratory evaluation and storage of biological specimens for genetic or molecular analysis in this trial/future use

Blood will be taken for analysis. Once the sites have been delivered to the local research facility, the samples will be collected, stored, and processed in compliance with local protocols. Blood shall be stored between 2 and 8°C before being treated in the designated time. Every sample collected for the research will have a patient identification code attached; otherwise, no personally identifying information will be included. Patients have the option to consent to the preservation of their samples in case they are needed for additional research.

Samples will be anonymously stored at a central location for a minimum of five years and a maximum of 10 years after the research is completed. Following that, they will be burned to ashes in compliance with regional customs and laws. The potential use of the stored samples is mentioned in the informed consent form. Any further use of the documents, nevertheless, will require approval from the institutional ethics council.

### Sample size calculation

Based on our power calculation, we aim to recruit 28 patients for both groups, with a total of 56 participants. Quadriceps muscle strength measured by the handheld dynamometer will be the primary outcome for sample size estimation. The effect size of the isometric quadriceps muscle strength has been determined as 0.34 in patients with knee OA [36]. A repeated-measures one-way analysis of variance (ANOVA) will be used to compare knee muscle strength, muscle mass, physical functional test, biomarkers, and questionnaire score for four timepoints (before the vitamin D supplementation (Baseline), at 3months, 6 months, and 12 months after the commencement of vitamin D supplementation). When the sample size is 23 per group with 4 repeated measurements, will have 80% of the power to detect at the 0.05 significance level. An additional 20% will be added to account for possible attrition. Thus, our sample size of 28 participants per group (total *n* = 56) will be sufficient to achieve our objective. The sample size was calculated using G*Power 3.0 software.

### Statistical analysis

Data in this study will be analyzed according to the intention-to-treat principle. Only the full analysis set and per-protocol set will be used for primary outcome analysis. The last observation carried forward (LOCF) will be used to impute the missing data by replacing it with the last observed value.

Quantitative variables will be expressed as mean ± standard deviation. Normality tests will be performed to determine whether the data is normally distributed. A one-way analysis of covariance (ANCOVA) tests with Bonferroni correction and with the baseline value as a covariate was conducted at 3m,6m, and 12m to determine the group effect on each parameter. Independent T-test (two-tailed) are used for multiple testing of continuous variables. Whereas the Chi-square test will be used to compare the proportions of categorical variables and to calculate the differences in the count data. A repeated measure analysis of variance (ANOVA) will be used to analyze the trend of changes in the scores among four-time points (Baseline, 3m, 6m, 12m) to determine the group×time effects. Post hoc analysis with Bonferroni correction will be conducted to explore the differences between baseline and 3m, baseline and 6m, 6m and 12m. The statistical analysis will be performed using commercialized statistical software (SPSS, version 29.0, IBM). All statistical significance is defined as P < 0.05.

Additional analyses will include subgroup analyses to estimate treatment effects for both female and male participants, normal BMI and overweight or obese participants.

### Interim analyses

Interim analysis will be performed when approximately 20% of our participants have completed follow-up assessments. The early findings will be presented at conference to promote our study.

### Plans to give access to the full protocol, participant-level data, and statistical code

The protocol has been uploaded on ClinicalTrials.gov (No.: NCT05981534). The data of this study will also be available from the principal investigator upon reasonable request.

### Oversight and monitoring

#### Composition of the coordinating center and trial steering committee

The coordination of various collaboration partners and the study design are under the purview of the primary investigator. The research team is made up of the trial steering committee, which is in charge of hiring, evaluating, gathering, and analyzing data.

#### Composition of the data monitoring committee, its role and reporting structure

The principal investigator and other investigators will supervise the data collection and storage procedures to ensure that the data is preserved and used in accordance with protocol. A statistician free from competing interests and the sponsor will inspect clinical data collected during the research period, assess interim analysis, and report back to the investigators for any necessary action.

#### Frequency and plans for auditing trial conduct

An independent research staff will conduct the annual review throughout the project period according to the requirement of the university.

#### Plans for communicating important protocol amendments to relevant parties (e.g. trial participants, ethical committees)

No plans to change the procedure now. Any changes to the protocol, however, must be reported and approved by the ethical committee prior to adoption by the principal investigator. Furthermore, notification will also be sent to the trial participants.

#### Trial status

The initial protocol was dated 09^th^ June 2022, and version 3 was dated 26^th^ February 2024. Reasons for revision were suggested by the ethics committee for the further justification of the required sample size.

Study recruitment started on 17 March 2024, and the recruitment is still ongoing.

## Discussion

Conservative therapies that target symptoms (e.g., pain, stiffness, and activity limitations), such as exercise, are recommended as first- and second-line treatments for knee osteoarthritis [37]. Patients with end-stage knee OA will be recommended to take the TKR as the viable option after the failure of conservative, non-surgical management [38]. However, TKR has shown to be effective in the management of knee OA symptoms and disability. This surgery may not be suitable for all patients as 20%-40% of patients reported dissatisfaction [39, 40], persistent symptoms [41], reduced lower limb muscle strength [42], impaired physical function such as walking performance and stair climbing postoperatively [43]. Consequently, there is a great need for minimally invasive, reversible treatments with high patient acceptance and excellent safety and efficacy to fill the large OA treatment gap.

Since the 1980s, when assays of 25-hydroxyvitamin D (25(OH)D) were developed as a marker of vitamin D status, multiple observational studies have supported the hypothesis of an inverse association between vitamin D status and muscle health [44]. Based on mainly observational data, it has been inscribed in existing studies that vitamin D improves lower limb function and muscle strength in the condition of low level of vitamin D [45, 46]. While limited studies were conducted to see if vitamin D supplementation on knee muscle strength, mass, and function in end-stage knee OA.

Patients on the waiting list for TKR usually experience excruciating pain and have significant functional impairment that may prevent them from conducting exercise. And longer delays before surgeries may result in deterioration in pain, functional performance and health-related quality of life, which could eventually affect post-surgery outcomes [47]. Considering the long waiting time for TKR, limited surgeries were performed in Hong Kong. An effective clinical non-surgical treatment should be provided for end-stage knee OA before they undergo the TKR. A well-designed, double-blinded randomized controlled trial could test if vitamin D has clinical effects and fill the gap of lack of alternative non-surgical treatment for end-stage knee OA with vitamin D insufficient or deficient on the waiting list.

This study started to recruit participants from 17^th^ March 2024. The success of this study will provide scientific evidence of whether the relatively cheap, and well-tolerated vitamin D supplement can improve quadriceps muscle strength, physical function, pain symptoms, and sarcopenia status of this increasingly large population for end-stage knee OA patients. The impact of this study is particularly strong given the long and increasing waiting time for TKR in Hong Kong.

## Supporting information

S1 File SPIRIT checklist. (PDF)

S2 File Initial and updated ethics approval. (PDF)

S3 File Chinese and English versions of consent forms. (PDF)

S4 File Approved protocol (Version 03, dated October 2023). (PDF)

S5 File Questionnaires. (PDF)

## Abbreviations

ADL: Function in daily living
ANCOVA: analysis of covariance
ANOVA: analysis of variance
BDNF: Brain-derived neurotrophic factor
CONSORT: Consolidated Standards of Reporting Trials
CREC: Clinical Research Ethics Committee
DXA: Dual Energy X-ray Absorptiometry
ELISA: enzyme-linked immunosorbent assay
FFQ: Food Frequency Questionnaire
FGF: Fibroblast growth factor
GMP: Good Manufacturing Practice
HA: Hospital Authority
IGF-1: Insulin-like growth factor 1
IL: Interleukin
IPAQ: International Physical Activity Questionnaire
KOOS: Knee Injury and Osteoarthritis Outcome Score
OA: Osteoarthritis
P3NP: Procollagen type III N-terminal peptide
QOL: Quality of Life
RCT: randomized controlled trial
SF-36: Short-Form 36
SPARC: Secreted protein acidic and rich in cysteine
SPIRIT: Standard Protocol Items: Recommendations for Interventional Trials
Sport/Rec: Function in Sport and Recreation
TKR: total knee replacement
TNNT1: C-terminal of troponin T1
WOMAC: Western Ontario and McMaster Universities Osteoarthritis Index
