# Supplementary material for: The effectiveness of vitamin D supplementation in patients with end-stage knee osteoarthritis: Study protocol for a double-blinded, randomized controlled trial
Source: PLoS One. 2024 Oct 21;19(10):e0309610. doi: 10.1371/journal.pone.0309610 (PMC11493297; doi:10.1371/journal.pone.0309610)
Supplement: S3 File — (PDF) [file pone.0309610.s003.pdf]

# Patient Information Sheet

‘Double-blinded randomized controlled trial investigating the effectiveness of vitamin D supplementation in patients with end-stage knee osteoarthritis (OA)’

## Introduction

You are invited to participate in a research study conducted by Professor Michael Tim-Yun Ong from the Department of Orthopaedics and Traumatology at the Chinese University of Hong Kong. OA knee is one of the most common musculoskeletal complaints, resulting in elderly patients living with severe pain limiting their daily activities. Many of these patients are homebound and therefore are at higher risk of vitamin D insufficiency. The inactivity is also a significant risk factor for sarcopenia. We are therefore in dire need to improve the well-being of this large and increasing group of patients as the capacity for operations remained limited.

## Description of the study

Vitamin D has long been recognized for its effect on musculoskeletal health and increasing attention has been focused for its effect on muscle function. The proposed project aims to set up a double-blinded RCT to compare the efficacies of vitamin D supplements, in addition to resistance exercise, against sarcopenia among end-stage OA knee patients with vitamin D insufficiency or deficiency. We hypothesized that insufficiency of vitamin D contributes to the poor muscle strength in patients with end-stage OA knees, more significantly in sarcopenic knee OA patients.

## Procedure

The duration of this study will be for two year. You will be invited to complete a set of questionnaires to evaluate your knee function, activity level, and a set of assessments regarding your muscle strength, volume and quality at baseline level,3-, 6-, 12- month post-interventions. A 6-month Vitamin D supplement (4000 IU/day) or placebo course will begin after baseline assessment. Your blood (5ml each) will be taken at baseline, and 3-, 6-, 12-month post-intervention. The whole procedure will not interfere with your normal medical care or rehabilitation program. There are no alternative treatments in this study. If you refused to take the Vitamin D course in this study, you will be excluded from the study and receive the normal medical care as discussed with your doctor.

## Risks and Benefits

There may be potential risks during exercise, but they are relatively low. Please contact the responsible investigators if you feel any discomfort. In the event of any injuries caused directly by the procedures of this study, our organization will offer appropriate medical treatment. Medical costs incurred by such treatment would be paid for by our organization but is limited to the amount not already covered by your insurance, government benefits or other responsible third parties. Participation in this study will not cause you to lose any rights to compensation according to existing laws. There are no direct benefits involved in this study, as the evidence supporting the immediate benefits of vitamin D supplementation for your condition has not been fully established. However, the current study may lead to the potential development of adjuvant treatments for knee osteoarthritis and sarcopenia.

## Voluntary Participation

Your participation is voluntary; this means you can choose to withdraw at any time without negative consequences.

Termination of Study

Although this is not generally expected, you should be aware that your study doctor may withdraw you from the study for any of the following reasons.

- Withdrawal is of your best interest in terms of health and welfare.
- You fail to follow the instructions given by your study doctor.
- You require a drug and/or treatments that is not allowed by the study.
- You become pregnant.
- You experience a serious adverse event and require treatment or observation, so that you study doctor deems inappropriate to continue the study.
- The study is being terminated by the investigators.

Information Protection

Your personal information and data will only be accessed by the principal investigator, researchers involved in the study, and the regulatory authorities. If the results of the study are published, your identity will remain confidential. The researcher will keep the information collected for at least **3 years** beyond the end of the study and the tissues collected will be stored in an established tissue bank at the Prince of Wales Hospital.

Contact person

If you have any inquiries about this study, please contact the principal investigator Professor Michael Tim Yun Ong at 35052083 or the co-investigator Miss Xiao Min Lu/ Qian Wen Wang at 26364171. You can also acquire your rights via The Joint Chinese University of Hong Kong – New Territories East Cluster Clinical Research Ethics Committee at 35053935.

**Statement of Consent**

I,____________________________(full name), consent to participate in the study 'Double-blinded randomized controlled trial investigating the effectiveness of vitamin D supplementation in patients with end-stage knee osteoarthritis (OA) '.

I have read the consent form, understand that the procedure and risks involved and have received answers to any questions I asked. In light of new findings, our team would like to study the underlying mechansim such as certain vitamin D pathway that affects the muscle quality. In such case, the blood samples would be re-evaluated for further investigation, like the measurement of other biomarkers. **I understood the nature of this study and agree that the information collected will be kept by the researcher for at least 3 years beyond the end of the study** and blood samples collected will be kept in an established tissue bank at Prince of Wales Hospital. I understand that the data collected will be published to the public and in peer-reviewed scientific paper anonymously.

Signature: ___________________

Name: ______________________

Date: _______________________

Signature of person obtaining consent: _____________________

Name of person obtaining consent: _________________________

Date: _______________________

*If you have any inquiries, you can contact the following persons for more information:
Principal investigator: Professor Ong Michael Tim Yun Tel: 35052083
Co-investigator: Miss Xiao Min Lu/ Qian Wen Wang Tel: 26364171
Joint CUHK-NTEC CREC Tel: 35053935

維他命 D 對改善終末期膝骨性關節炎患者的肌肉力量及功能雙盲對照試驗研究

簡介
我們誠邀您參加一項由香港中文大學骨科和創傷醫學系的王添欣教授主持的一項研究，以了解維他命D對改善患終末期的膝骨性關節炎患者的肌肉力量及功能的效果。膝關節炎是最常見的肌肉骨骼疾病之一，老年患者經常主訴感到劇烈疼痛，從而導致他們的日常活動受到限制。 許多患者都因活動受限長居在家，因此缺乏維生素 D 的風險更高。 缺少活動也是導致肌少症的一個重要風險因素。 因此，由於增加手術能力仍然有限，我們極需改善這一龐大且不斷增加的患者群體的健康情況。

研究背景
一直以來，維生素D因其對肌肉骨骼健康的影響而受到關注，而它對肌肉功能的影響也越來越引起研究者注意。這一專案旨在建立一個雙盲對照試驗，比較維生素D補充劑和抗阻訓練對患有維生素 D 不足或缺乏的終末期膝骨關節炎患者肌肉疏鬆症的功效。 我們假設，維生素D不足是導致終末期膝骨關節炎患者肌肉力量低下的原因，在同時患有肌少症的患者中更為明顯。

計劃程序
這項研究的期限為兩年。 您將被邀請完成一套問卷，以評估您的膝關節功能、活動水平，以及在基線、維生素 D 干預後 3 個月、6 個月、12 個月的肌肉力量、體積和品質方面的評估。基線評估後，將開始為期 6 個月的維生素 D 補充劑(4000IU/天)，或安慰劑干預。 同時，將在基線、干預後 3 個月、6 個月以及 12 個月抽取你的血液（每次 5 毫升）。 整個過程不會干擾您的正常醫療護理或康復計劃。 在這項研究中，沒有其他的治療方法。 如果您拒絕接受本研究中的維生素 D 補充劑計劃，您不會被納入研究範圍， 並會依舊按照醫生的指導進行正常醫療護理。

風險與利益
與本研究所相關的風險很低。參加者需需要遵循指示攝取補充劑，否則可能會有健康損害的風險。如參加者感到任何不適，請聯絡研究人員。如果閣下因為本研究的程序直接受傷，我們會提供適當的治療。該治療的費用將會由我們支付，但只會包括不受保險、政府福利或其他第三方賠償的金額。參與本研究並不會使閣下失去任何取得賠償的法定權利。由於維他命 D 對您當前情況的益處尚無研究證明，本研究沒有任何直接的利益。研究結果可能會對研發針對膝骨性關節炎患者的肌肉力量及功能的特定治療有幫助。

自願參與
您的參與完全為自願性質。您參與這項研究與否將不影響您所應得的醫療服務及法律權利。即使在您參與這項研究後，您仍可在任何時候退出，而無需任何理由。如果你決定參與此研究，你需要在此同意書上簽署。研究人員會幫你安排時間進行研究所涉及的各項檢查，請你在檢查的當日準時到達檢查地點。如果您不能應約，請盡早聯絡研究人員，以便更改覆診時間。

終止研究的參與

在少數的情況下，研究人員有可能因以下原因終止閣下在此研究的參與：

- 終止參與合符個下健康的最大利益。
- 閣下未能按照醫生或研究人員的指示進行研究程序。
- 閣下需要接受與本研究相抵觸的藥物或治療。
- 閣下於研究期當中懷孕。
- 閣下經歷嚴重不良事件並需要接受治療或醫學監察，以使研究人員認為閣下不適合繼續參與此研究。
- 本研究被研究人員終止。

個人資料保護

參加者的資料絕對保密，只有研究負責人，副負責人及參與項目的研究助理可以參閱。在不違反研究對象的私隱的情況下，香港中文大學－新界東醫院聯網臨床研究倫理聯席委員會將被授權查閱記錄以核查臨床試驗程序和數據將被授權查閱記錄以核查臨床試驗程序和數據。如研究試驗的結果會公佈，參加者的身份將嚴格保密。如研究試驗的結果會公佈，參加者的身份將嚴格保密。所收集的信息將由研究人員保留至研究結束後至少 3 年，所收集的組織樣本將會有部分供威爾斯親王醫院組織庫作研究用途。

聯絡人

如對此研究計劃有任何查詢，請直接聯絡研究負責人或副負責人。你亦可以透過香港中文大學－新界東醫院聯網 臨床研究倫理聯席委員會，查詢你的權益。

負責人: 王添欣教授 電話: 35052083
副負責人: 盧曉敏小姐/王倩雯小姐 電話: 26364171
香港中文大學－新界東醫院聯網 臨床研究倫理聯席委員會 電話: 3505 3935

「維他命 D 對改善終末期膝骨性關節炎患者的肌肉力量及功能雙盲對照試驗研究」

**研究計劃 - 同意書**

本人 ________________________ ，同意參與名為「維他命 D 對改善患終末期的膝骨性關節炎患者的肌肉力量及功能雙盲對照試驗研究」。

本人已知悉有關程序及風險，并對於此項計劃的疑問都得到了解答。鑒於可能會有新的發現，我們的團隊希望進一步研究潛在的機制，如研究维他命 D 代謝途徑是如何影響肌肉質量的。在這種情况下，血液樣本將被重新評估以進行進一步調查，如測量其他生物標志物。本人同意所收集的信息將由研究人員保留至研究結束後至少 3 年，所收集的血液樣本將會保留與威爾斯親王醫院的組織庫，並了解所收集的數據將以匿名方式發布給公眾和同行評審的科學論文。

簽名: __________________________

姓名: ____________________________

日期: _____________________________

徵求同意人員簽名: ______________________

徵求同意人員姓名: __________________________

日期: _____________________________

*如閣下想了解更多詳情，可致電以下人士查詢:

負責人: 王添欣教授 電話: 35052083

副負責人: 盧曉敏小姐/王倩雯小姐 電話: 26364171

香港中文大學－新界東醫院聯網 臨床研究倫理聯席委員會 電話: 3505 3935
