# Supplementary material for: The effectiveness of vitamin D supplementation in patients with end-stage knee osteoarthritis: Study protocol for a double-blinded, randomized controlled trial
Source: PLoS One. 2024 Oct 21;19(10):e0309610. doi: 10.1371/journal.pone.0309610 (PMC11493297; doi:10.1371/journal.pone.0309610)
Supplement: S4 File — (PDF) [file pone.0309610.s004.pdf]

a) **Title:**

Double-blinded randomized controlled trial investigating the effectiveness of vitamin D supplementation in patients with end-stage knee osteoarthritis (OA)

b) **Introduction:**

Osteoarthritis (OA) knee is one of the commonest chronic degenerative conditions. It causes disability in elderlies due to pain and stiffness. The prevalence of radiologic knee osteoarthritis increases in proportion to age, reaching an astounding 64.1% for patients who are over 60 years of age. In 2021, there were over 26,000 patients on the Hospital Authority (HA) waiting list for knee total knee replacement (TKR) and with only 4300 TKRs performed, the nominal waiting time for TKR was almost 89 months. With an ageing population, the number of patients suffering from end-stage OA knees waiting for surgery is expected to increase significantly. Although TKR is the definitive treatment for patients with end-stage OA, other interventions can be offered to these patients to improve their overall health and function during their long wait for TKR.

<u>**Impact of Knee OA on Localized and Generalized Muscle Health**</u>

Patients with end-stage knee OA often adopt a sedentary lifestyle to avoid joint pain and stiffness. This can have an adverse effect on muscle function both **locally around the knees, as well as on generalized muscle health.**

Local: The muscle disuse due to OA knees can lead to quadriceps muscle atrophy and the subsequent knee-related muscle weakness can be a cause for exacerbating the symptoms of OA knees. A systematic review reported an increased risk of symptomatic knee OA in persons with knee extensor muscle weakness (1) and it is a factor for knee OA progression (2). Pre-operative quadriceps muscle strength and function has been shown to correlate with post-operative outcome (3). Quadriceps muscle strengthening exercise is a key component for the management of OA knee, but the response to exercise is often variable.

Generalized: "Sarcopenic Knee OA": Ageing and inactivity leads to a progressive loss of muscle mass and strength until an abnormally low level, termed as "sarcopenia" (4). Sarcopenic elderlies are more likely to develop deterioration of functional outcomes and higher mortality resulting in socioeconomic and healthcare burdens (5). Diagnosis of sarcopenia follows the criteria set by the Asian Working Group for Sarcopenia (AWGS) (6). For Chinese community-dwelling older adults, the pooled prevalence of sarcopenia was 14%(7), whereas in our previous study investigating the prevalence of sarcopenia in end-stage OA showed that **32.8% with severe knee OA patients also suffered sarcopenia and these patients showed a slower recovery after undergoing TKR**(8). A recent study categorised "sarcopenic knee OA" patients as a new subgroup, with higher risk of falling and associated risk factors than patients with sarcopenia or knee OA alone (9). Resistance exercise has been shown to improve muscle mass, strength, and gait speed among sarcopenic patients (10). However, the individual responses to exercise are variable (11).

Individual responses (intrinsic factors) to exercise training may account for patients failing to improve despite resistance exercises. As the waiting list for TKR in Hong Kong is long, and the capacity to increase the number of TKR annually is limited, it is of great importance to investigate for these intrinsic factors and enhance the effect of exercise training, in order to improve the musculoskeletal health of this increasingly large number of patients during the long wait for TKR. Patients with end stage OA knees will have more pain, leading to decreased mobility, higher risk of sarcopenia and vitamin D insufficiency that would warrant attention.

<u>**Potential Role of Vitamin D**</u>

Low vitamin D can adversely affect cartilage thickness and study suggested that low serum vitamin D is associated with increased radiographic knee OA progression (12). A systematic review concluded that vitamin D supplements can improve pain and function in patients with knee OA (13).

Muscle

Vitamin D has long been recognized for its effect on musculoskeletal health and increasing attention has been focused for its effect on muscle function. Vitamin D have a direct effect on muscle hypertrophy by acting on specific vitamin D receptors (VDRs) on myocytes, and sufficient levels of vitamin D in patients have been found to correlate with an increase in the size, number, and strength of muscle fibres. Vitamin D also seems to exert beneficial effects by its interplay with myokines such as myostatin and irisin. It has been shown that elderly individuals with vitamin D deficiency are susceptible to sarcopenia and there are biological, clinical, and epidemiological evidence supporting the association between vitamin D and an increased risk of sarcopenia in older people (14). The study also showed that muscle nuclear VDR was increased by 30% and augmented muscle fibre size by 10% in elderly females (mean age of 78 years) **taking vitamin D orally at a rate of 100 µg/day (4000 IU/day) for 4 months** (14).

Vitamin D level (deficiency, insufficiency, or sufficiency)

Vitamin D deficiency is defined as serum levels of 25(OH)D less than 20 ng/mL at baseline. Insufficiency from 20 to <30 ng/mL and ≥30 ng/mL is considered as vitamin D sufficiency(15). In the Chinese population aged over 65, 30.6% of the population studied presented with vitamin D deficiency (25(OH)D <20 ng/mL)(16). Up to 62% of elderly patients with OA was found to have vitamin D insufficiency (17), and the risk of low vitamin D is particularly high when compared with normal population as many of these patients are confined indoor due to the decreased mobility limited by pain(18).

Supporting Evidence

Animal: In our animal study using mice, we have shown the level of vitamin D decreases with advancing age, and the levels of vitamin D correlated positively with lean muscle mass (Appendix 1 Fig 1b)and the grip strength (Fig 1c).

Clinical: Our previous published study investigating patients pending knee replacements has reflected that **32.8% with severe knee OA patients also suffered sarcopenia, compared to 14% in elderly general population**, and these patients were found to have a slower function recovery after operation(8,10).

In our pilot study of **9 patients with sarcopenia, 7 (78%) of the patients were found to have vitamin D insufficiency/deficiency (5 deficient, 2 insufficient)**, with only 2 (22%) who was vitamin D sufficient. Patients with vitamin D deficiency was found to have a significantly lower gait speed, reflective of a poorer lower limb function.

Clinical Significance

OA knee is one of the most common musculoskeletal complaints, resulting in elderly patients living with severe pain limiting their daily activities. Many of these patients are homebound and therefore are at higher risk of vitamin D insufficiency. The inactivity is also a significant risk factor for sarcopenia. We are therefore in dire need to improve the well-being of this large and increasing group of patients as the capacity for operations remained limited. With a well-conducted randomized controlled trial (RCT), we aim to provide evidence for Vitamin D supplementation to improve muscle status and knee symptoms in end stage OA knee patients.

Novelty

This is the first double-blinded RCT investigating the effect of vitamin D supplements for knee OA patients. This can provide evidence of whether the relatively cheap, and well-tolerated vitamin D supplement can improve quadriceps muscle strength, the physical function and pain symptoms of this

increasingly large population. The impact of this study is particularly strong given the long and increasing waiting time for TKR in Hong Kong.

c) **Aims and Hypotheses to be Tested:**

Aims

The proposed project aims to set up a double-blinded RCT to investigate the effect of vitamin D supplementation for knee muscle strength, physical function, pain symptoms and, sarcopenia status among end-stage OA knee patients with vitamin D insufficiency or deficiency.

1. To investigate the effect of vitamin D supplementation for muscle strength in end-stage OA knee patients.
2. To investigate the effect of vitamin D supplementation for physical function symptoms of OA knees in end-stage OA knee patients.
3. To investigate whether the effect of vitamin D supplementation to improve the status of sarcopenia

Hypothesis

We hypothesized that insufficiency of vitamin D contributes to the poor muscle strength in patients with end-stage OA knees We believe that the vitamin D intervention group will have:

1. Improved muscle strength
2. Improved physical function and symptoms of OA knees
3. Improved the status of sarcopenia.

d) **Plan of Investigation:**

This will be a **double-blinded RCT** investigating the **effect of vitamin D supplements** for **patients** with **end-stage knee OA**. The study will be a follow-up study with assessment at baseline, 3 and 6-month after the commencement of vitamin D supplement, and 6-month after the interventional period. This study is under the registration process on ClinicalTrials.gov. We will conduct the study in compliance with the Declaration of Helsinki.

The primary outcome assessment

Improvement of muscle strength reflected by quadriceps and hamstring muscle strength. Hand grip strength will also be measured.

The secondary outcome assessment

Improvement of physical function by 6-meter timed gait test and 5-time chair stand test.
Improvement of muscle mass by Dual Energy X-ray Absorptiometry (DXA).
Improvement of symptoms of OA knees by the Western Ontario and McMaster Universities Osteoarthritis Index (WOMAC) and the Knee Injury and Osteoarthritis Outcome Score (KOOS).

Other assessments

Biochemical: serum levels of myokines and vitamin D.

(i) Subjects

56 patients with end-stage knee OA will be recruited from the Li Ka Shing Orthopaedic Specialist clinic at the Prince of Wales Hospital (PWH) Hong Kong. The inclusion and exclusion criteria are as follows:

Inclusion Criteria:

(1) Male and female patients aged over 50 with end-stage knee OA
(2) Patients are waiting for TKR at Prince of Wales Hospital
(3) Walk unaided for 6 meters
(4) Able to comply with the assessments and has given oral and written consent
(5) Patients with vitamin D insufficiency and deficiency at the baseline measurement (25(OH)D <30 ng/mL)

Exclusion Criteria

(1) Patients with connective tissue disorders or myositis condition
(2) History of any Hip & Knee surgery
(3) Patients with malnutrition assessed by Mini-Nutritional assessment.
(4) Patients with acute immobility (i.e., post hip fracture or post-acute hospital admission)
(5) Patient scheduled for TKR within 6 months
(6) Patients already taking vitamin D supplements
(7) Patients with a known contraindication to vitamin D treatment (such as allergy)
(8) Patients who have renal impairment with glomerular filtration rate (eGFR) < 30 ml/minute

<u>Sample Size Estimation</u>

Based on our power calculation, we aim to recruit 28 patients for both groups with a total of 56 participants.

Quadriceps muscle strength will be the primary outcome for sample size estimation. The effect size of the isometric quadriceps muscle strength has been determined as 0.34 in patients with knee OA (21). A repeated ANOVA will be used to compare muscle strength, muscle mass, physical functional test, biomarkers and questionnaire score before the vitamin D supplementation and at 3 months, 6 months after the commencement of vitamin D supplementation, and at 6 months post intervention. When the sample size is 23 per group with4 repeated measurements, a one-way analysis of variance will have 95% of power to detect at the 0.05 level. An additional 20% will be added to account for possible attrition. Thus, 56 (28x2) subjects will be recruited in total.

In previous studies related to OA knee, we have positive response rate from patients around 86% to join our studies, given that the patients were already screened from the inclusion criteria. We currently have over 2300 patients on the waiting list for knee replacement surgery. We will be able to complete recruitment within 12 months.

(ii) Methods

Oral and written consents will be obtained from individuals who agree to participate in the study. The recruitment period will last for 12 months and the whole project period is 2 years in total. Basic demographics, sarcopenia assessment and Outcome Measurement Questionnaires will be carried out. The patients will be advised to avoid taking supplements and keep a record of medication intake throughout the study period.

Basic Demographics

Pre-operation baseline demographics will be collected for inter-patient comparisons:

1. Gender
2. Age
3. Height, Weight and Body Mass Index
4. Duration of Symptomatic Knee OA
5. Ethnicity

Sarcopenia Assessment

Diagnosis of sarcopenia will base on the criteria recommended by Asian Working Group for Sarcopenia (AWGS). If a low gait speed (<0.8m/s) or low handgrip strength is detected (<26kg for male and <18kg for female according to AWGS) and a low muscle mass is confirmed by DXA (<7kg/m 2 in male and <5.4 kg/m 2 for female according to AWGS), the patient is diagnosed as sarcopenia.

Group assignments

Patients will be randomized into two treatment groups.

Group 1: Vitamin D supplement

Group 2: Placebo

Vitamin D Therapy

We will use 4000 IU/day, for 6 months adapted from previous study (22). All study tablets including the placebo will be manufactured according to Good Manufacturing Practice (GMP) guidelines for quality assurance.

Randomization

A 1:1 randomization (23 subjects per arm, 28 with 20% accounted for drop-out rate) will be used. To guarantee allocation concealment, an independent statistician not involved in this study will pre-generate random binary numbers to indicate the group assignment. The generated numbers will be printed and stored in a sealed envelope respectively. Sealed envelopes with group assignment codes will be distributed consecutively; subjects will break the seal after completing baseline assessments.

Maintenance and measurement of compliance

Any complications and other complaints from the participants will be monitored and taken care of by medical officers. Any adverse event or problems arise during the study will be reported directly to the ethnic committee in the institution. In addition, participants are allowed to quit the study at any time for any reason; if so, they will be asked whether they wish to be followed up according

to the trial schedule. A cut-off level of 80% will be used to categorize the compliant and non-compliant groups.

Muscle Strength

1. Quadriceps and hamstring muscle strength assessment by hand-held dynamometer

Hand-held dynamometer microFET2 (Hoggan Scientific, Salt Lake City UT, USA) will be used to assess lower limb strength and power. Assessment of isometric muscle strength and power will be performed with the participants in a seated position to assess knee extensors and knee flexors. All tests will involve maximal voluntary isometric contractions. Both limbs will be assessed to record side-to-side difference. Two trials were recorded for each muscle group.

2. Handgrip Dynamometer

The handgrip dynamometer will be used to test for the patients' handgrip strength. The handgrip dynamometer is an instrument measuring patients' maximum isometric strength of the hand and forearm muscles. The handle of the dynamometer will be adjusted as the finger is at 90 ° whilst the dynamometer is being held. The measurement will be repeated three times and the average calculated for the dominant hand (Hand dominance will be determined by observing the patient's writing hand when signing the informed consent).

Physical Function

1. 6-meter Timed Walking Gait Test

The 10-meter timed walking test is a well-established test to assess gait speed. However, due to space limitations and the exhaustive nature of the test for patients with OA, the 6-meter test has been documented to be a valid and reliable substitute. Patients will be asked to walk a straight line of 6 meters where the time taken to complete the distance will be measured. (<7.5 seconds is normal).

2. Chair stand test

The chair stand test is a reliable test for assessment for low limb strength in patients. Patients will be asked to sit on a solid chair with arms on shoulders and feet with shoulder width apart. They will be instructed to do 1-2 repetitions to become familiar with the test with the test and perform 5 reputations for two trials. Time will be recorded.

Muscle Mass

1. Dual Energy X-ray Absorptiometry (DXA)

The radiation for one session (20 minutes) for sarcopenia assessment is less than 25μSv, which is within the safe range. After every DXA scan, two copies will be printed that show the patients' body composition, bone mineral density, fat percentage, body mass index, and most importantly for this study, lean muscle mass.

Symptoms of OA knees

1. Western Ontario and McMaster Universities Osteoarthritis Index (WOMAC)

It is a questionnaire that measures a patient's pain, stiffness and physical function and can be summed up into a score out of 96. A high score indicates a more disabled participant (Appendix 3).

2. Knee Injury and Osteoarthritis Outcome Score e (KOOS)

The Knee injury and Osteoarthritis Outcome Score (KOOS) was to evaluate symptoms and function in patients with knee injury and osteoarthritis with five separately scored subscales: Pain, other Symptoms, Function in daily living (ADL), Function in Sport and Recreation (Sport/Rec), and knee-related Quality of Life (QOL). (Appendix 4).

Other Outcome Measurement

Questionnaires

Health-related quality of life will be assessed through two validated and reliable questionnaires (i.e. SF-12 and IPAQ).

1. Short-Form 36 (SF-36)

12 questions to measure a patient's functional health and well-being from a patient's point of view. It is a reliable and validated measure that summarises the patients' physical and mental health (Appendix 5).

2. International Physical Activity Questionnaire (IPAQ)

Assesses and monitors a patient's physical activity and inactivity level. This instrument sums up a patient's activity level per week into three categories, Category 1 Inactivity, category 2 minimally active and category 3 HEPA active (Appendix 6).

3. Nutritional intake questionnaire

Evaluation of habitual dietary intake will be based on retrospective means of assessment for the past twelve months. A Food Frequency Questionnaire (FFQ) previously validated with data obtained in the Hong Kong Adult Dietary Survey in 1995 as described previously will be used(Appendix 7). Daily dietary intake of Vitamin D and calcium will be evaluated by the Food Processor Nutrition Analysis and Fitness software version 7.9 (Esha Research, Salem, USA), with incorporation of local food composition based on food composition table from China(23).

4.Sunlight exposure questionnaire

The Chinese version of the sunlight exposure questionnaire will be used in this study and has proven to be adequate for measurement of lifetime sunlight exposure among Hong Kong Chinese women (24).

Biochemical Assays

Blood samples will be taken under non-fasting conditions. The serum obtained will be immediately stored at -80˚C until analysis.

1. Serum myokine evaluation

Blood taking (5 ml) will be performed. The serum will be prepared by centrifugation and kept in a -80º freezer until use. Quantitative analysis for myokines and proteins related to muscle metabolism will be performed by Human Myokine Magnetic Bead Panel (Millipore) with Bioplex-200 bead-based suspension assay system (LKSIHS core facilities), or enzyme-linked immunosorbent assay (ELISA). These include Brain-derived neurotrophic factor (BDNF), Fibroblast growth factor-21 (FGF-21), Interleukin-6 (IL-6), IL-15, Irisin, Myostatin (MSTN)/GDF8, Insulin-like growth factor 1 (IGF-1), FGF-2, IL-8, Follistatin, Musclin, Myonectin, Decorin, Meteorinlike, Osteopontin, Secreted protein acidic and rich in cysteine (SPARC), Klotho,

Procollagen type III N-terminal peptide (P3NP), and C-terminal of troponin T1 (TNNT1).

2. Serum vitamin D assays

Serum 25(OH)Vit-D levels will be measured by commercial 25(OH) Vitamin D ELISA kit (Abcam ab213966) according to the manufacturer's instruction, providing the quantitative determination of 25(OH) Vitamin D3 and 25(OH) Vitamin D2. Sensitivity: 1.98 ng/ml (Range: 0.5 ng/ml - 1010 ng/ml).

(iii) Study design

Patients will be assessed on knee quadriceps and hamstring strength, handgrip strength, gait speed, chair stand test, outcome measurement questionnaires and blood test for serum vitamin D and myokines level at recruitment, after3,6 months of vitamin D supplement, and after the interventional period (at 6 months). The muscle mass will be assessed with DXA at recruitment and end of the interventional period and 6 month post intervention (Appendix 2).

(iv) Data processing and analysis

Based on our power calculation, we aim to recruit 28 patients for both groups with a total of 56 participants. Analyses will be performed as intention-to-treat, defined as all participants randomized, regardless of whether they finished the full study protocol. The spread of data will be tested for normality. Parametric and non-parametric analysis will then be used to determine for normally distributed and non-normally distributed data. For normally distributed data, Students' t-

test and Chi square test will be used to determine the difference in the distribution of continuous and categorical data, respectively. While for non-normally distributed data, Mann Whitney test and Wilcoxen Rank Tests will be used to determine the difference in the distribution of continuous and categorical data, respectively. Repeated ANOVA will be used to analyze the trend of changes in the scores with two factors of groups and time.P value of <0.05 will be considered statistically significant.

(v) Potential pitfalls and contingency plans

Patients that are potentially intolerable/allergic of high dose vitamin D supplements or have pre-existing medical condition that could lead to potential contraindications will not be included in our study during screening. If the patient record did not indicate any potential adverse reaction to the supplement but experience such a situation during intervention, the patient would be immediately removed from the study in their best interest to safeguard their health. Study personnel will reach out the patients regularly to ensure the patients is not experiencing any discomfort due to the supplements. Study personnel would indicate and explain to patients during recruitment about the intervention duration and frequency to stress the importance of compliance, accompanied with frequent reminders to participants. In the case of patient non-compliance, study personnel would remove the patient from the study only when multiple failed attempts to contact and convince the patient occurs.

If the number of patients recruited is low, as our team work in both Prince of Wales Hospital and the the Alice Ho Miu Ling Nethersole Hospital (AHNH) in Tai Po, we can also include patients from the AHNH, which is a joint centre performing around 500 TKR annually.

[Word Count: 3998]

e) Existing Facilities:

The proposed study will be conducted in the Prince of Wales Hospital (PWH). Subject inclusion, follow-up visit, clinical examination, and questionnaire filling will be conducted in the Out-patient Clinic of Orthopaedics and Traumatology, which is located at the 1/F of Li Ka Shing Specialist Clinic (North Wing) in the hospital. Processing of blood samples, myokine measurement will be conducted in the Lee Hysan Clinical Laboratories located at 9-11/F of Li Ka Shing Institute of Health Sciences (LiHS) in PWH. A full gear of centralized research facilities is available (http://www.cuhk.edu.hk/med/crl/equipment.html), including liquid nitrogen tanks in cryostorage room (room 1103), cryostat in Histology Laboratory (room 906), large capacity refrigerated centrifuges in Centrifuge Laboratory (room 1009), and microplate reader (room 1002). Our Musculoskeletal Research Laboratory (room 1003) is also well-equipped with various machines necessary for the proposed study, including bench-top refrigerated centrifuges, deep freezers, staining station, and microscopes. Details of the facilities can be found on the website (http://www.ort.cuhk.edu.hk/research-facilities.html).

f) Justification of Requirements:

1) Staff cost: A research assistant (RA) will be required throughout the study period to assist PA to work on all parts, including recruitment, informed consent, subject randomization, data collection (conducting functional outcome measurements, filling of questionnaires during the visits). He/she will also help in data processing and analysis, preparation of reports, conference abstracts and manuscripts. Considering the time required to recruit 48 subjects, the workload of the clinicians and research

assistant, the duration of intervention on each participant, and the longest follow-up of 12 months after commencement of treatment, the RA will work for 24 months at full time.

2) DXA scans: DXA scan allows the team to analyze body composition in a breakdown of bone mass, fat tissue, and muscle mass creating a powerful feedback loop. It would help track the body composition changes of research participants throughout the study period by comparing index of different research time point. Cost of equipment usage includes the use of DXA. One session of DXA on assessing of muscle/lean mass for indication of sarcopenia would cost HK$1,200. Assessment of osteoporosis using clinical DXA bone scans will also be taken as well as the total body scan of muscle using the same DXA machine at a time. One session of DXA on measurement of bone density for osteoporosis indication would cost another HK$1,200 each. For each of the 48 patients recruited two sessions of DXA are necessary. Therefore, a total of 192 sessions of DXA is required. The total amount of expense on equipment usage will be HK$ 230,400.

3) Muscle Biopsy: Muscle biopsy signify the present of certain type of muscle fibre that be indicative of muscle strength of estimation in a person.

4) Serological Measurement (myokines and Vd levels): Blood samples will be collected from all subjects at 5-time points. The cost will include the multiplex reagent kits and the service provided by Core Facilities Molecular Biology of the Li Ka Shing Institute of Health Sciences in CUHK. ELISA kits were budgeted to measure proteins that are not included in the multiplex assays.

5) Vitamin D and placebo capsules: According to the study design, subjects will take either Vitamin D or placebo capsules (Maltitol) for 4000 IU/day for 26 weeks. The estimated cost is HK$ 78612, including certificates of analysis

6) Printing and Documentation cost: Printing cost for informed consent and questionnaire for all follow-up time points will be included. External hard disk (2TB) will be required for storage of participants' data.

7) Conference: Travel expenses, accommodation (international conference), the registration fee is budgeted to support 1 local conference and 1 international conference.

8) Publication: For publication costs, the cost for submission fee/article processing fee/publication fee is budgeted for 1 paper in a peer-reviewed journal.

g) Plan to Disseminate Research Findings to End Users:

During the long waiting time for TKR surgery in HK, our proposed treatment strategy will be a practical and essential component of prehab before the surgery. We will aim to implement this therapeutic strategy in our clinical practice within two years if our hypothesis is tested----we will propose the supplements to be administered to patients that are diagnosed with sarcopenia and on the waiting list for TKR. As our hypothesis, we hope to find improved muscle status, along with reduced knee symptoms on these patients. For patients undergoing TKR, our proposed treatment strategy can potentially achieve a better and faster recovery post operatively.

At a later stage with clearly proven results, we would suggest promoting it among patients who are first diagnosed with OA to improve muscle status, reduce the knee symptoms, and to improve their quality of life.

h) Key References:

1. Marks R. Muscle and muscle mechanisms as possible factors leading to osteoarthritis. SM J Orthop. 2015;1(2):1008.

2. Javadian Y, Adabi M, Heidari B, Babaei M, Firouzjahi A, Ghahhari BY, Hajian-Tilaki K. Quadriceps Muscle Strength Correlates With Serum Vitamin D and Knee Pain in Knee Osteoarthritis. Clin J Pain.

2017 Jan;33(1):67-70.
3. Devasenapathy N, Maddison R, Malhotra R, Zodepy S, Sharma S, Belavy DL. Preoperative Quadriceps Muscle Strength and Functional Ability Predict Performance-Based Outcomes 6 Months After Total Knee Arthroplasty: A Systematic Review. Phys Ther. 2019 Jan 1;99(1):46-61. doi: 10.1093/ptj/pzy118. PMID: 30329137.
4. Cruz-Jentoft AJ, Bahat G, Bauer J, Boirie Y, Bruyère O, Cederholm T, et al. Sarcopenia: revised European consensus on definition and diagnosis. Age and ageing. 2019;48(1):16-31.
5. Ho AW, Lee MM, Chan EW, Ng HM, Lee C, Ng W, et al. Prevalence of pre-sarcopenia and sarcopenia in Hong Kong Chinese geriatric patients with hip fracture and its correlation with different factors. Hong Kong Med J. 2016;22(1):23-9.
6. Chen L-K, Woo J, Assantachai P, Auyeung T-W, Chou M-Y, Iijima K, et al. Asian Working Group for Sarcopenia: 2019 consensus update on sarcopenia diagnosis and treatment. Journal of the American Medical Directors Association. 2020;21(3):300-7. e2.
7. Xin C, Sun X, Lu L, Shan L. Prevalence of sarcopenia in older Chinese adults: a systematic review and meta-analysis. *BMJ Open.* (2021) 11:e041879.
8. Ho KK, Lau LC, Chau WW, Poon Q, Chung KY, Wong RM. End-stage knee osteoarthritis with and without sarcopenia and the effect of knee arthroplasty - a prospective cohort study. BMC Geriatr. 2021 Jan 4;21(1):2.
9. Iijima H, Aoyama T. Increased recurrent falls experience in older adults with coexisting of sarcopenia and knee osteoarthritis: a cross-sectional study. BMC Geriatr. 2021 Dec 15;21(1):698.
10. Liao Y, Peng Z, Chen L, Zhang Y, Cheng Q, Nüssler AK, et al. Prospective views for whey protein and/or resistance training against age-related sarcopenia. Aging and disease. 2019;10(1):157.
11. Nomura T, Kawae T, Kataoka H, Ikeda Y. Assessment of lower extremity muscle mass, muscle strength, and exercise therapy in elderly patients with diabetes mellitus. Environmental health and preventive medicine. 2018;23(1):1-7.
12. Malas, F.Ü., Kara, M., Aktekin, L. *et al.* Does vitamin D affect femoral cartilage thickness? An ultrasonographic study. *Clin Rheumatol* **33,** 1331–1334 (2014).
13. Zhao, ZX., He, Y., Peng, LH. *et al.* Does vitamin D improve symptomatic and structural outcomes in knee osteoarthritis? A systematic review and meta-analysis. *Aging Clin Exp Res* **33,** 2393–2403 (2021).
14. Uchitomi R, Oyabu M, Kamei Y. Vitamin D and Sarcopenia: Potential of Vitamin D Supplementation in Sarcopenia Prevention and Treatment. Nutrients. 2020;12(10):3189.
15. Gallagher JC, Sai AJ. Vitamin D insufficiency, deficiency, and bone health. Oxford University Press; 2010.
16. Wei, J., Zhu, A. & Ji, J.S. A Comparison Study of Vitamin D Deficiency among Older Adults in China and the United States. Sci Rep 9, 19713 (2019).
17. Haroon M, Bond U, Quillinan N, Phelan MJ, Regan MJ. The prevalence of vitamin D deficiency in consecutive new patients seen over a 6-month period in general rheumatology clinics. Clin Rheumatol. 2011 Jun;30(6):789-94. doi: 10.1007/s10067-010-1659-0. Epub 2010 Dec 24. PMID: 21184246.
18. Jansen JA, Haddad FS. High prevalence of vitamin D deficiency in elderly patients with advanced osteoarthritis scheduled for total knee replacement associated with poorer preoperative functional state. Ann R Coll Surg Engl. 2013;95(8):569-572.
19. Nerhus TK, Heir S, Thornes E, Madsen JE, Ekeland A. Time-dependent improvement in functional outcome following LCS rotating platform knee replacement. Acta Orthop. 2010;81(6):727-732.
20. Reginster JY, Beaudart C, Al-Daghri N, Avouac B, Bauer J, Bere N, Bruyère O, Cerreta F, Cesari

M, Rosa MM, Cooper C, Cruz Jentoft AJ, Dennison E, Geerinck A, Gielen E, Landi F, Laslop A, Maggi S, Prieto Yerro MC, Rizzoli R, Sundseth H, Sieber C, Trombetti A, Vellas B, Veronese N, Visser M, Vlaskovska M, Fielding RA. Update on the ESCEO recommendation for the conduct of clinical trials for drugs aiming at the treatment of sarcopenia in older adults. Aging Clin Exp Res. 2021 Jan;33(1):3-17.

21. Kean CO, Birmingham TB, Garland SJ, Bryant DM, Giffin JR. Minimal detectable change in quadriceps strength and voluntary muscle activation in patients with knee osteoarthritis. Arch Phys Med Rehabil. 2010 Sep;91(9):1447-51.

22. Wyon MA, Koutedakis Y, Wolman R, Nevill AM, Allen N. The influence of winter vitamin D supplementation on muscle function and injury occurrence in elite ballet dancers: a controlled study. Journal of science and medicine in sport. 2014;17(1):8-12.

23. Yang Y, Wang G, Pan X. China Food Composition 2004. Beijing: Beijing Medical University Publishing House; 2005.

24. Wu S, Ho SC, Lam TP, et al. Development and validation of a lifetime exposure questionnaire for use among Chinese populations. Sci Rep 2013;3:2793.
