# Supplementary material for: The effectiveness of vitamin D supplementation in patients with end-stage knee osteoarthritis: Study protocol for a double-blinded, randomized controlled trial
Source: PLoS One. 2024 Oct 21;19(10):e0309610. doi: 10.1371/journal.pone.0309610 (PMC11493297; doi:10.1371/journal.pone.0309610)
Supplement: S5 File — (PDF) [file pone.0309610.s005.pdf]

WOMAC Osteoarthritis index					
**關節疼痛多寡程度(總分 20 分)**					
在過去一個禮拜,當您從事下列活動時有哪些項目是會使您的關節感到疼痛?	完全不痛	輕微的痛	中度的痛	嚴重的痛	非常嚴重的痛
1.走在平坦的路上，您的關節有多痛?	0	1	2	3	4
2.上下樓梯時，您的關節有多痛?	0	1	2	3	4
3.晚上睡覺時, 您的關節有多痛?	0	1	2	3	4
4.坐或躺，您的關節有多痛?	0	1	2	3	4
5.筆直站立時，您的關節有多痛?	0	1	2	3	4
**關節僵硬程度(總分 8 分)**	完全不僵硬	輕微僵硬	中度僵硬	嚴重僵硬	非常嚴重僵硬
1.早晨剛起床時,您的關節有多僵硬?	0	1	2	3	4
2.約莫傍晚時分,若您坐一下、躺一下或休息一下之後,您的關節有多僵硬?	0	1	2	3	4
**身體功能(總分 68 分)** 在過去一個禮拜，您因關節炎而導致活動的程度?	完全不困難	有一點困難	中度困難	相當困難	困難到極點
1.下樓時，您感到有多困難?	0	1	2	3	4
2.上樓時，您感到有多困難?	0	1	2	3	4
3.從椅子上站起來時，您感到有多困難?	0	1	2	3	4

4.站的時候，您感到有多困難?	0	1	2	3	4
5.彎腰時，您感到有多困難?	0	1	2	3	4
6.走在平坦的路上，您感到有多困難?	0	1	2	3	4
7.上車及下車時，您感到有多困難?	0	1	2	3	4
8.逛街買東西時，您感到有多困難?	0	1	2	3	4
9.穿上襪子時，您感到有多困難?	0	1	2	3	4
10.從床上起身時，您感到有多困難?	0	1	2	3	4
11 脫掉襪子時，您感到有多困難?	0	1	2	3	4
12.躺在床上時，您感到有多困難?	0	1	2	3	4
13 近出浴室洗澡時，您感到有多困難?	0	1	2	3	4
14 坐的時候，您感到有多困難?	0	1	2	3	4
15.上廁所時，您感到有多困難?	0	1	2	3	4
16 做粗重家事時，您感到有多困難?	0	1	2	3	4
17.做簡單家事時，您感到有多困難?	0	1	2	3	4

Hong Kong Chinese KOOS KNEE SURVEY

姓名:
出生日期: ____(日)/____(月)/____(年)
填寫問卷日期: ____(日)/____(月)/____(年)

說明：

這個調查會詢問一些關於你的膝關節問題。這些資料將會幫助我們了解你對膝關節的感覺以及你進行日常活動的能力。在回答每條問題時，請在合適的方格內以☑顯示，每題只能選一個答案。如果你不是很確定怎樣回答一條問題，請盡量選擇一個你認為最好的答案。

症狀

請想想你在過去一星期膝關節的症狀，然後回答這些問題。

S1 你的膝關節會否腫脹？

不會	偶爾	有時	時常	經常
☐	☐	☐	☐	☐

S2 當膝關節活動時，你會否感到磨擦，或聽到膝關節發出任何聲音？

不會	偶爾	有時	時常	經常
☐	☐	☐	☐	☐

S3 你的膝關節在活動時有否卡住？

不會	偶爾	有時	時常	經常
☐	☐	☐	☐	☐

S4 你能完全伸直膝關節嗎？

經常	時常	有時	偶爾	不能
☐	☐	☐	☐	☐

S5 你能完全彎曲膝關節嗎？

經常	時常	有時	偶爾	不能
☐	☐	☐	☐	☐

僵硬

請想想你在過去一星期膝關節僵硬的程度，然後回答這些問題。

S6 早上剛醒來時，你的膝關節有多僵硬？

不會	少許	普通	嚴重	極度
☐	☐	☐	☐	☐

S7 坐下、躺下後你的膝關節有多僵硬？

不會	少許	普通	嚴重	極度
☐	☐	☐	☐	☐

疼痛

P1　你多常感到膝痛？

從不	每月一次	每週一次	每日一次	經常
☐	☐	☐	☐	☐

過去一星期，你在進行以下活動時會感到什麼程度的痛楚？

P2　以膝關節為中心扭動或轉動身體

沒有痛楚	輕微疼痛	頗為疼痛	非常疼痛	極度疼痛
☐	☐	☐	☐	☐

P3　完全伸直膝關節

沒有痛楚	輕微疼痛	頗為疼痛	非常疼痛	極度疼痛
☐	☐	☐	☐	☐

P4　完全彎曲膝關節

沒有痛楚	輕微疼痛	頗為疼痛	非常疼痛	極度疼痛
☐	☐	☐	☐	☐

P5　在平地步行

沒有痛楚	輕微疼痛	頗為疼痛	非常疼痛	極度疼痛
☐	☐	☐	☐	☐

P6　上或下樓梯

沒有痛楚	輕微疼痛	頗為疼痛	非常疼痛	極度疼痛
☐	☐	☐	☐	☐

P7　晚上就寢

沒有痛楚	輕微疼痛	頗為疼痛	非常疼痛	極度疼痛
☐	☐	☐	☐	☐

P8　坐下或躺下

沒有痛楚	輕微疼痛	頗為疼痛	非常疼痛	極度疼痛
☐	☐	☐	☐	☐

P9　挺直站立

沒有痛楚	輕微疼痛	頗為疼痛	非常疼痛	極度疼痛
☐	☐	☐	☐	☐

日常生活

過去一星期，你在進行以下活動時膝關節使你感到有多困難？

A1　下樓梯

沒有困難	少許困難	頗大困難	非常困難	極大困難
☐	☐	☐	☐	☐

過去一星期，你在進行以下活動時膝關節使你感到有多困難？

A2 上樓梯

沒有困難	少許困難	頗大困難	非常困難	極大困難
☐	☐	☐	☐	☐

A3 從坐姿站起

沒有困難	少許困難	頗大困難	非常困難	極大困難
☐	☐	☐	☐	☐

A4 站立

沒有困難	少許困難	頗大困難	非常困難	極大困難
☐	☐	☐	☐	☐

A5 彎腰至地／在地上檢起物品

沒有困難	少許困難	頗大困難	非常困難	極大困難
☐	☐	☐	☐	☐

A6 在平地步行

沒有困難	少許困難	頗大困難	非常困難	極大困難
☐	☐	☐	☐	☐

A7 上車／下車

沒有困難	少許困難	頗大困難	非常困難	極大困難
☐	☐	☐	☐	☐

A8 逛街購物

沒有困難	少許困難	頗大困難	非常困難	極大困難
☐	☐	☐	☐	☐

A9 穿襪子／絲襪

沒有困難	少許困難	頗大困難	非常困難	極大困難
☐	☐	☐	☐	☐

A10 起床

沒有困難	少許困難	頗大困難	非常困難	極大困難
☐	☐	☐	☐	☐

A11 脫掉襪子／絲襪

沒有困難	少許困難	頗大困難	非常困難	極大困難
☐	☐	☐	☐	☐

A12 躺在床上 （轉身，維持膝關節姿勢）

沒有困難	少許困難	頗大困難	非常困難	極大困難
☐	☐	☐	☐	☐

過去一星期，你在進行以下活動時膝關節使你感到有多困難？

A13 進出浴缸

沒有困難	少許困難	頗大困難	非常困難	極大困難
☐	☐	☐	☐	☐

A14 坐下

沒有困難	少許困難	頗大困難	非常困難	極大困難
☐	☐	☐	☐	☐

A15 坐上／離開坐廁

沒有困難	少許困難	頗大困難	非常困難	極大困難
☐	☐	☐	☐	☐

A16 做粗重的家務 （剷雪、擦地等）

沒有困難	少許困難	頗大困難	非常困難	極大困難
☐	☐	☐	☐	☐

A17 做輕巧的家務 （煮食、除塵等）

沒有困難	少許困難	頗大困難	非常困難	極大困難
☐	☐	☐	☐	☐

運動與休閒

過去一星期，你在進行以下活動時膝關節有否使你感到困難？

SP1 蹲坐

沒有困難	少許困難	頗大困難	非常困難	極大困難
☐	☐	☐	☐	☐

SP2 跑步

沒有困難	少許困難	頗大困難	非常困難	極大困難
☐	☐	☐	☐	☐

SP3 跳躍

沒有困難	少許困難	頗大困難	非常困難	極大困難
☐	☐	☐	☐	☐

SP4 轉動／扭動受傷的膝關節

沒有困難	少許困難	頗大困難	非常困難	極大困難
☐	☐	☐	☐	☐

SP5 跪下

沒有困難	少許困難	頗大困難	非常困難	極大困難
☐	☐	☐	☐	☐

膝關節對生活質量的影響

Q1　你多常注意到你的膝關節問題？

從不	每月一次	每星期一次	每日一次	經常
☐	☐	☐	☐	☐

Q2　你有否改變生活模式來避免一些有機會傷及膝關節的活動？

沒有改變	少許改變	頗大改變	很大改變	完全改變
☐	☐	☐	☐	☐

Q3　你是否對你的膝關節缺乏信心？

完全沒有	少許	普通	嚴重	極度
☐	☐	☐	☐	☐

Q4　總括來說，你的膝關節對你的生活造成多大影響？

完全沒有	少許	頗大	非常大	極大
☐	☐	☐	☐	☐

～非常感謝您完成了這份調查中所有的問題～

The Chinese (Hong Kong) SF-36 Health Survey

簡明健康狀況調查表 – 香港中文譯本

Eight HRQOL Domains (八項健康生活質數範疇)

Physical functioning - 體能

Role-physical - 日常活動

Bodily Pain - 身體痛楚

General Health - 整體健康

Vitality - 精力

Social Functioning - 社交活動

Role-emotional - 日常活動 (心理健康的影響)

Mental Health - 心理健康

簡明健康狀況調查表 (SF-36)

1. 總括來說，你認為你的健康狀況是:

(只圈出一個答案)

極好 ········· 1

很好 ········· 2

好 ········· 3

一般 ········· 4

差 ········· 5

2. 和一年前比較，你認為你目前全面的健康狀況如何?

(只圈出一個答案)

比一年前好多了 ········· 1

比一年前好一些 ········· 2

和一年前差不多 ········· 3

比一年前差一些 ········· 4

比一年前差多了 ········· 5

3. 下列各項是日常生活中可能進行的活動。以你目前的健康狀況，你在進行這些活動時，有沒有受到時間限制? 如有的話，程度如何?

(每項只圈出一個答案)

活動	有很大限制	有一點限制	沒有任何限制
a. 劇烈運動，比如跑步，搬重物，或參加劇烈的體育活動	1	2	3
b. 中等強度的活動，比如搬桌子，使用吸塵器清潔地面，玩保齡球或打太極拳	1	2	3
c. 提起或攜帶蔬菜，食品或雜貨	1	2	3
d. 上幾層樓梯	1	2	3
e. 上一層樓梯	1	2	3
f. 彎腰，跪下或俯身	1	2	3
g. 步行十條街以上 (一公里)	1	2	3
h. 步行幾條街 (幾百米)	1	2	3
i. 步行一條街 (一百米)	1	2	3
j. 自己洗澡或穿衣服	1	2	3

4. 在過去四個星期裏，你在工作或其他日常生活中，會不會因為身體健康的原因而遇到下列的問題?

(每項只圈出一個答案)

	會	不會
a 減少了工作或其他活動時間	1	2
b 實際做完的比想做的要少	1	2
c 工作或其他活動的種類受到限制	1	2
d 進行工作或其他活動時有困難 (比如覺得更為吃力)	1	2

5. 在過去的四個星期裡，你在工作或其他日常生活中，會不會由於情緒方面的原因 (比如感到沮喪或焦慮) 遇到下列的問題?

(每項只圈出一個答案)

	會	不會
a 減少了工作或其他日常活動的時間	1	2
b 實際做完的比想做的要少	1	2
c 工作時或從事其他活動時不如往常細心了	1	2

6. 在過去四個星期裡，你的身體健康或情緒問題在多大程度上妨礙了你與家人、朋友、鄰居或社團的日常社交活動?

(只圈出一個答案)

毫無妨礙 ………………………………………………… 1

有很少妨礙 ………………………………………………… 2

有一些妨礙 ………………………………………………… 3

有較大妨礙 ………………………………………………… 4

有極大妨礙 ………………………………………………… 5

7. 在過去四個星期裡，你私身體有沒有疼痛， 如果有的話，疼痛到什麼程度?

(只圈出一個答案)

完全沒有 ………………………………………………… 1

很輕微 ………………………………………………… 2

輕微 ………………………………………………… 3

有一些 ………………………………………………… 4

劇烈 ………………………………………………… 5

非常劇烈 ………………………………………………… 6

8. 在過去四個星期裡，你身體上的疼痛對你的日常工作 (包括上班和家務) 有多大影響?

(只圈出一個答案)

毫無影響 ………………………………………………………… 1

有很少影響 ……………………………………………………… 2

有一些影響 ……………………………………………………… 3

有較大影響 ……………………………………………………… 4

有極大影響 ……………………………………………………… 5

9. 下列問題是有關你在過去星期裡你覺得怎樣和你其他的情況。針對每一個問題，請選擇一個最接近你感覺的答案。

在過去四個星期裡有多少時間:

(每項只圈出一個答案)

	常常如此	大部分時間	相當多時間	有時	偶爾	從來沒有
a. 你覺得充滿活力 ?	1	2	3	4	5	6
b. 你覺得精神非常緊張?	1	2	3	4	5	6
c. 你覺得情緒低落，以致於沒有任何事能使你高興起來?	1	2	3	4	5	6
d. 你感到心平氣和?	1	2	3	4	5	6
e. 你感到精力充足?	1	2	3	4	5	6
f. 你覺得心情不好，悶悶不樂 ?	1	2	3	4	5	6
g. 你感到筋疲力盡?	1	2	3	4	5	6
h. 你是個快樂的人?	1	2	3	4	5	6
i. 你覺得疲倦?	1	2	3	4	5	6

10. 在過去四個星期裡，有多少時間由於你的身體健康或情緒問題妨礙了你的社交活動 (比如探親、妨友等)？

(只圈出一個答案)

常常有妨礙 ………………………………………………………… 1

大部分時間有妨礙 ………………………………………………………… 2

有時有妨礙 ………………………………………………………… 3

偶爾有妨礙 ………………………………………………………… 4

完全沒有妨礙 ………………………………………………………… 5

11. 如果用下列的句子來形容你，你認為有多正確?

(每項只圈出一個答案)

	肯定對	大致對	不知道	大致不對	肯定不對
a 你好像比別人更容易生病	1	2	3	4	5
b 你好像所有你認識的人一樣健康	1	2	3	4	5
c 你覺得自己的身體狀況會變壞	1	2	3	4	5
d 你的健康極好	1	2	3	4	5

國際身體活動問卷

我們想要了解民眾在日常生活中，所做身體活動的狀況。想請教您的是： 您在過去七天中花在身體活動的時間，包括工作、做家事、整理庭院／陽台、 交通，及您在休閒時間所做的娛樂、運動等活動中所花的時間。就算您認為自己不愛動，也請您回答每一個問題。

回想過去七天中，所有您做過強而有力、及適度的活動。

強而有力的身體活動：這些活動會讓您的身體感覺很累，呼吸會比平常快很多，活動時對話會很喘或呼吸感困難。這類活動的強度跟慢跑差不多，**不過持續時間須多於十分鐘，才可被計算在內**。

適度的活動：這些活動會讓您覺得身體有點累，呼吸會比平常快一些，活動時還可以舒服的對話。這類活動的強度跟快步走差不多，**不過持續時間須多於十分鐘，才可被計算在內**。

1. 過去七天中，您花多少天做**強而有力的身體活動, 像是提重物、有氧運動或快騎單車?**
   - ☐ 有，共有____天
   - ☐ 沒有**強而有力的身體活動**，請跳答問題 3

2. 承上題，在參與**強而有力的身體活動**的日子，你通常花多少時間做強而有力的身體活動?
   - ☐ **每天**__________小時以及________分鐘
   - ☐ 不知道/不確定

3. 想一想最近七天你所做過的所有**適度的活動**，你花多少天做**適度的活動**，像是正常速度下騎單車，但**不包括走路**?
   - ☐ 有，共有____天
   - ☐ 沒有**適度的活動**，請跳答問題 5

4. 承上題，在參與**適度的活動**的日子，你通常花多少時間做**適度的活動**?
   - ☐ **每天**__________小時以及________分鐘
   - ☐ 不知道/不確定

5. 想一想最近七天你花多少天在**走路**(至少持續十分鐘)，包括工作、在家、從某地到某到、娛樂、遊戲或休閒時走路?
   - ☐ 有，共有____天
   - ☐ 沒有**走路**，請跳答問題 7

6. 承上題，在有**走路**的日子，你通常花多少時間走路?
   - ☐ **每天**__________小時以及________分鐘
   - ☐ 不知道/不確定

7. 在最近連續七個非假日時間(扣除週六與週日)，你平均每天花多少時間在坐著，包括花在工作、家裡、做作業以及休閒時的**坐著**?
   - ☐ **每天**__________小時以及________分鐘
   - ☐ 不知道/不確定

問卷調查完畢，謝謝你的參與。

Please insert Label
Serial Number:
Name: 姓名:
DOB: Sex/Age:
HKID:
Date:

香港中文大學
The Chinese University of Hong Kong

飲食問卷調查
Food Frequency Questionnaire

Have you checked the participant's name? 核對参加者姓名: ☐ Yes/有

Interviewer's Number 訪問員編號: | H | K | | |

五穀類 Grains

食物種類 Type of Food	編碼 Code	過去一年的次數 How Often Within the Past Year? 從未 Never	一年幾次 A Few Times per Year	一月一次 Once per Month	一月二至三次 2 – 3 Times per Month	一星期一次 Once per Week	一星期二次 Twice per Week	一星期三至四次 3 – 4 Times per Week	一星期五至六次 5 – 6 Times per Week	每日 Every day	每次多少 How much each time?	參考份量 Reference Portion	Code	Data Entry	
		0	6	12	30	52	104	182	286	365					
米飯 Rice	274											1 bowl = 200g	38184		8
紅米/糙米飯 Red/Brown Rice												1 bowl = 200g	38010		8
稀飯 Soft Rice	277											1 bowl = 200g	S20		8
清粥 Congee	276											1 bowl = 200g	P33		8
麵/烏冬 Wheat Noodles/Udon	270											1 bowl = 200g	38273		8
即食麵 Instant Noodles	272											1 bowl = 100g	P35 (dried)		8
米粉 Rice Vermicelli	280											1 bowl = 200g	38146		8
通心粉 Macaroni	269											1 bowl = 200g	38258		8
意大利粉 Pasta	273											1 plate = 100g	38262		8
麥皮 Oatmeal	286											1 bowl = 200g	40072		8
粟米片 Corn Flakes	311											1 box = 25g	40195		8
香甜玉米片 Frosties	314											1 box = 25g	40217		8
饅頭 Chinese Steam Buns (Mann-Tau)	2023											1 piece = 50g	P10		8
咸包 Plain rolls	254 (roll)											1 piece = 70g	71351		8
白方包 White Breads	251 (w/ crust)											1 slice = 50g	71255		8
去皮白方包 White Breads (without edges)	251 (no curst)											1 slice = 30g	71255		8
全麥麵包 Whole Wheat Breads	253 (w/ crust)											1 slice = 40g	42014		8
去皮全麥麵包 Whole Wheat Breads (without edges)	253 (no curst)											1 slice = 30g	42014		8
甜包 Sweet Rolls	255											1 piece = 70g	P212		8
其他 Others															

Remarks: 8=gram, 11=ml

蔬菜類/豆類 Vegetables & Beans

食物種類 Type of Food	編碼 Code	過去一年的次數 How Often Within the Past Year?									每次多少 How much each time?	參考份量 Reference Portion	Code	Data Entry	
		從未 Never	一年幾次 A Few Times per Year	一月一次 Once per Month	一月二至三次 2 – 3 Times per Month	一星期一次 Once per Week	一星期二次 Twice per Week	一星期三至四次 3 – 4 Times per Week	一星期五至六次 5 – 6 Times per Week	每日 Every day					
		0	6	12	30	52	104	182	286	365					
菜心 Choy Sum	1202											1 plate = 100g	V62		8
白菜 Bok Choy	1201 (p)											1 plate = 100g	5237		8
芥蘭 Chinese Kale	1245											1 plate = 100g	7909		8
西蘭花 Broccoli	1207											1 plate = 50g	5028		8
椰菜花 Cauliflowers	1208											1 plate =50g	5051		8
莧菜 Chinese Spinach	1211											1 plate = 100g	V5		8
西洋菜 Watercress	1237											1 plate = 100g	5222		8
菠菜 Spinach	1270											1 plate = 100g	5147		8
通菜 Water Spinach	1209											1 plate = 100g	V63		8
韭菜 Chinese Chives	1247											1 plate = 100g	V2		8
豆苗 Pea Shoots	1242											1 plate = 100g	V21		8
椰菜 Cabbages	1204											1 plate = 100g	5038		8
黃芽白 Celery Cabbages	1203											1 plate = 100g	V16		8
生菜 Lettuce	1205											1 plate = 100g	5083		8
蘆筍 Asparagus	1238											1 plate =50g	5003		8
西芹 Celery	1271											1 plate =50g	5056		8
鮮黃豆 Fresh Soybeans	1244											1 serving = 50g	L10		8
硬豆腐 Tofu, Hard	1552											1 serving = 50g	7960		8
布包豆腐 Tofu, Soft	1552 (cube)											1 cube = 300g	90630		8
腐竹 Dried Tofu Sheets	1553											1 serving = 50g	L2		8
油炸豆腐 Deep Fried Tofu	1576											1 piece = 60g	7520		8
豆腐泡 Deep Fried Tofu Pockets	1554											1 piece = 16 g	L13		8
粉絲 (乾) Mung Bean Noodles												1 piece = 50g	90200		8
腐皮 Tofu Skin	1556											1 serving = 50g	L20		8

食物種類 Type of Food	編碼 Code	過去一年的次數 How Often Within the Past Year? 從未 Never 0	一年幾次 A Few Times per Year 6	一月一次 Once per Month 12	一月二至三次 2 – 3 Times per Month 30	一星期一次 Once per Week 52	一星期二次 Twice per Week 104	一星期三至四次 3 – 4 Times per Week 182	一星期五至六次 5 – 6 Times per Week 286	每日 Every day 365	每次多少 How much each time?	參考份量 Reference Portion	Code	Data Entry	
齋雞 Vegetarian Chicken	2651											3 pieces = 50g	P4		8
麵筋 Wheat Gluten	284											1 serving = 50g	P38		8
綠豆芽 Mungbean Sprouts	1222											1 plate = 100g	5021		8
黃豆芽 Soybean Sprouts	1223											1 plate = 100g	5459		8
焗茄豆 Baked Beans	1551 (p)											1 serving = 50g	7038		8
紅豆(乾) Red Bean (Dried)	1560											1 serving = 25g	7113		8
眉豆(乾) Black Eye Peas, dried	1558 (p)											1 serving = 50g	7017		8
四季豆/甜豆 Snap Beans	1243											1 plate = 50g	5011		8
荷蘭豆 Snow Peas	1230											1 plate = 100g	5666		8
青豆 Green Peas	1215 (p)											1 serving = 50g	7230		8
蠶豆 Broad Beans	1240											1 serving = 50g	7027		8
豆角 String Beans	1213											1 serving = 50g	V35		8
洋蔥 Onions	1226											1 serving = 50g	5108		8
紅蘿蔔 Carrots	1216 (p)											1 serving = 50g	5047		8
青蘿蔔 Turnips, Green												1 serving = 50g	6006		8
白蘿蔔 Turnips, White												1 serving = 50g	6001		8
粉葛 Chinese Radish	1246											1 serving = 50g	V45		8
蕃薯 Sweet Potatoes	1251											1 serving = 50g	5155		8
薯仔 Potatoes	1224											1 serving = 50g	5136		8
南瓜 Pumpkins	1253											1 serving = 100g	5396		8
蓮藕 Lotus Roots	1250											1 serving = 50g	5392		8
馬蹄 Water Chestnuts	1249											7 pieces = 50g	5386		8
竹筍 Bamboo Shoots	1241											1 serving = 50g	5249		8
節瓜 Hairy Melons	1227											1 serving = 100g	V61		8
青瓜 Cucumbers	1228											1 serving = 100g	7920		8

食物種類<br>Type of Food	編碼<br>Code	過去一年的次數<br>How Often Within the Past Year?									每次多少<br>How much each time?	參考份量<br>Reference Portion	Code	Data Entry	
		從未<br>Never	一年幾次<br>A Few Times per Year	一月一次<br>Once per Month	一月二至三次<br>2 – 3 Times per Month	一星期一次<br>Once per Week	一星期二次<br>Twice per Week	一星期三至四次<br>3 – 4 Times per Week	一星期五至六次<br>5 – 6 Times per Week	每日<br>Every day					
		0	6	12	30	52	104	182	286	365					
苦瓜<br>Bitter Melons	1248											1 serving = 100g	V7		8
冬瓜<br>Winter Melons	1212											1 serving = 100g	3911		8
蕃茄<br>Tomatoes	1221											1 serving = 100g	5170		8
紅椒/黃椒<br>Red/YellowCapsicum/pepper	1274											1 serving = 100g	5278		8
青椒<br>Green Capsicum	1273											1 serving = 100g	5661		8
新鮮粟米<br>Sweet Corns	1218											1 serving = 50g	5380		8
粟米粒<br>Canned Corns	1219											1 serving = 50g	5066		8
絲瓜<br>Angled Loofah	1252											1 serving = 100g	V8		8
茄子/矮瓜<br>Eggplants	1239											1 serving = 100g	5072		8
新鮮菇類<br>Fresh Mushrooms	1275											1 serving = 100g	5092		8
乾冬菇<br>Dried Mushrooms	1450											12 pieces = 25g	5383		8
罐頭菇類-草菇/蘑菇<br>Canned Mushroom	1453											8 pieces = 100g<br>3 pieces = 50g	7952		8
白木耳/雲耳/雪耳(熟)<br>White Fungus	1458											1 serving = 55g	V26		8
木耳 (乾)<br>Wood Fungus	1455											1 serving = 10g	V42		8
髮菜 (乾)<br>Black Moss	1454											1 serving = 10g	V12		8
炸菜<br>Preserved Radish	1501											6 slices = 50g	P62		8
冬菜<br>Preserved Vegetables	1503											1 tbsp = 5 g	P63		8
雪菜<br>Preserved Greens	1502											1 tbsp = 5 g	V9		8
栗子<br>Chestnut	1566											1 piece = 10g	4647		8
腰果<br>Cashew Nuts	1564											1 serving = 35g	4622		8
花生<br>Peanut	1568<br>(p)											1 serving = 25g	4763		8
其他<br>Others															8

水果類 Fruits

食物種類 Type of Food	編碼 Code	過去一年的次數 How Often Within the Past Year?									每次多少 How much each time?	參考份量 Reference Portion	Code	Data Entry	
		從未 Never	一年幾次 A Few Times per Year	一月一次 Once per Month	一月二至三次 2 – 3 Times per Month	一星期一次 Once per Week	一星期二次 Twice per Week	一星期三至四次 3 – 4 Times per Week	一星期五至六次 5 – 6 Times per Week	每日 Every day					
		0	6	12	30	52	104	182	286	365					
橙 Oranges	1726 (m)											1 piece = 150g	3228		8
西柚 Grapefruits	1706											1 piece = 240g	3820		8
蘋果 Apples	1701 (m)											1 piece = 150g	3000		8
梨 Pears	1717											1 piece = 122g	3272		8
香蕉 Bananas	1704 (m)											1 piece = 120g	3020		8
蜜瓜 Honeydew Melons	1714											1 serving = 100g	3080		8
西瓜 Watermelon	1729											1 serving = 100g	3142		8
菠蘿 Pineapples	1724											1 slice = 70g	3111		8
士多啤梨 Strawberries	1734											4 pieces = 100g	3134		8
藍莓 Blueberries												1 cup = 145g	3029		8
桃 Peaches	1721 (m)											1 piece = 200g	3096		8
芒果 Mangos	1713											1 piece = 200g	3221		8
柿 Persimmons	1728											1 piece = 100g	F16		8
奇異果 Kiwi fruits	1731											1 piece = 75g	3356		8
杏 Apricots	1702											1 piece = 35g	F12		8
西梅 Prunes	1720											2 pieces = 100g	F7		8
提子 Grapes	3082 (m)											10 pieces = 100g	71090		8
荔枝 Lychee	1711											10 pieces = 100g	3257		8
龍眼 Longans	1712											10 pieces = 30g	3254		8
車厘子 Cherries	1705 (l)											10 pieces = 100g	3036		8
木瓜 Papayas	1716											1 serving = 400g	3171		8
柚子(沙田柚) Pomelo	1733											1 piece = 70g	3758		8
檸檬 Lemons	1710											1 piece = 100g	F14		8

食物種類<br>Type of Food	編碼<br>Code	過去一年的次數<br>How Often Within the Past Year?<br>從未<br>Never	一年幾次<br>A Few Times per Year	一月一次<br>Once per Month	一月二至三次<br>2 – 3 Times per Month	一星期一次<br>Once per Week	一星期二次<br>Twice per Week	一星期三至四次<br>3 – 4 Times per Week	一星期五至六次<br>5 – 6 Times per Week	每日<br>Every day	每次多少<br>How much each time?	參考份量<br>Reference Portion	Code	Data Entry	
		0	6	12	30	52	104	182	286	365					
糖漿雜果<br>Fruit Cocktail in Syrup	1740											1 serving = 30g	3045		8
杏甫<br>Dried Apricot	1703											10 pieces = 20g	3013		8
西梅乾<br>Dried Prunes	1725											1 piece = 7g	3126		8
葡萄乾<br>Raisins	1709 (match box)											1 small box = 40g	3129		8
紅黑棗<br>Dried Dates	1732											15 pieces = 20g	F13		8
火龍果<br>Dragon Fruit												1 piece = 350g (中)	F10		8
榴槤<br>Durian												1 piece = 100g	3975		8
山竹<br>Mangosteens												1 piece = 50g	3520		8
其他<br>Others															
其他<br>Others															
其他<br>Others															

肉類 Meats

食物種類<br>Type of Food	編碼<br>Code	過去一年的次數<br>How Often Within the Past Year?<br>從未<br>Never	一年幾次<br>A Few Times per Year	一月一次<br>Once per Month	一月二至三次<br>2 – 3 Times per Month	一星期一次<br>Once per Week	一星期二次<br>Twice per Week	一星期三至四次<br>3 – 4 Times per Week	一星期五至六次<br>5 – 6 Times per Week	每日<br>Every day	每次多少<br>How much each time?	参考份量<br>Reference Portion	Code	Data Entry	
		0	6	12	30	52	104	182	286	365					
瘦叉燒/瘦肉<br>BBQ pork/Pork, Lean	580											6 pieces = 50g	12239		8
半肥瘦豬肉<br>Pork, Lean & Fat												6 pieces = 50g	12043		8
半肥瘦叉燒<br>BBQ Pork, Lean & Fat	581											5 pieces = 50g	M34		8
瘦排骨<br>Spare Ribs, Lean	569											4 pieces = 50g	12309		8
半肥瘦排骨<br>Spare Ribs, Lean & Fat	570											4 pieces = 50g	12010		8
瘦燒肉<br>Roast Pork, Lean	566 (1pc)											1 piece = 25g	12027		8
半肥瘦燒肉<br>Roast Pork, Lean & Fat (with 24% fat)	585											1 piece = 25g	12045		8
豬扒<br>Pork Chop	567											1 piece = 100g	12261		8
牛肉<br>Beef Flank	650											6 pieces = 50g	10956		8
肥牛<br>Beef, Lean & Fat												4 pieces = 50g	10265		8
牛扒<br>Beef Sirloin	551											1 piece = 100g	10926		8
牛腩<br>Briskets	562											4 pieces = 50g	10830		8
碎牛肉<br>Ground beef												1 tbsp = 20g	58124		8
牛舌<br>Ox Tongue	561											6 pieces = 50g	M055		8
雞，有皮<br>Chicken, with skin	602 (p)											1 serving = 400g	15086		8
雞，去皮<br>Chicken, without Skin	601 (p)											1 serving = 400g	15087		8
雞柳<br>Chicken Strips	612											6 pieces = 50g	15004		8
雞中翼<br>Chicken wing, mid section	604 (m)											1 piece = 30g	15045		8
雞全翼<br>Chicken Wing, Whole	604 (w)											1 piece = 100g	15045		8
雞脾<br>Chicken Thigh	608											1 piece = 100g	15043		8

食物種類 Type of Food	編碼 Code	過去一年的次數 How Often Within the Past Year? 從未 Never	一年幾次 A Few Times per Year	一月一次 Once per Month	一月二至三次 2 – 3 Times per Month	一星期一次 Once per Week	一星期二次 Twice per Week	一星期三至四次 3 – 4 Times per Week	一星期五至六次 5 – 6 Times per Week	每日 Every day	每次多少 How much each time?	參考份量 Reference Portion	Code	Data Entry	
		0	6	12	30	52	104	182	286	365					
燒鵝，有皮 Roast Goose, w/ skin	623											1 serving = 400g	14003		8
燒鵝，去皮 Roast Goose, no skin	624											1 serving = 400g	14002		8
燒鴨，有皮 Roast Duck, w/ skin	620											1 serving = 400g	14001		8
燒鴨，去皮 Roast Duck, no skin	621											1 serving = 400g	14000		8
羊肉 Lamb	640											6 pieces = 50g	13510		8
乳鴿 Baby Pigeons	630											1/4 piece = 50g	M078		8
雞肝 Chicken Liver	610											6 slices = 50g	15215		8
雞心 Chicken Heart	617											6 slices = 50g	M053		8
豬肝 Pig Liver	571											6 slices = 50g	12013		8
豬心 Pig Heart	587											6 slices = 50g	M054		8
豬腰 Pig Kidneys	586											6 slices = 50g	M056		8
牛什 Beef Offal	2015											6 slices = 50g	M071		8
腸仔 Hot Dog Sausage	578											1 roll = 50g	58029		8
大紅腸 Big Red Sausage	577											6 slices = 50g	M19		8
中國臘腸 Chinese Sausage	574											1 roll = 50g	P80		8
中國潤腸 Chinese liver sausage	591											1 roll = 50g	13083		8
腊鴨髀 Duck Thigh, Dried, Preserved & Salted	620											1 serving = 50g	M057		8
腊肉 Pork, Dried, Preserved & Salted	589											1 serving = 25g	M057		8
火腿 Ham	575											2 pieces = 50g	57878		8
午餐肉 Canned luncheon Meat	572											2 pieces = 50g	11913		8
煙肉 Bacon												2 slices = 15g	12000		8
牛丸 Beef Ball												5 pieces = 100g	P128		8
豬肉丸 Pork Ball												5 pieces = 100g	12099		8
漢堡扒 Hamburger Patties	552 (l)											1 piece = 70g	58123		8

魚類及海產類 Fishes & Seafood

食物種類<br>Type of Food	編碼<br>Code	過去一年的次數<br>How Often Within the Past Year?<br>從未<br>Never<br>0	一年幾次<br>A Few Times per Year<br>6	一月一次<br>Once per Month<br>12	一月二至三次<br>2 – 3 Times per Month<br>30	一星期一次<br>Once per Week<br>52	一星期二次<br>Twice per Week<br>104	一星期三至四次<br>3 – 4 Times per Week<br>182	一星期五至六次<br>5 – 6 Times per Week<br>286	每日<br>Every day<br>365	每次多少<br>How much each time?	参考份量<br>Reference Portion	Code	Data Entry	
鯇魚/烏頭<br>Grass Fish	952<br>(p)											1 serving = 200g	M43		8
大頭魚<br>Big Head Fish	988											1 serving = 200g	M069		8
鯪魚<br>Mud Carp	985											1 serving = 200g	M36		8
黃鱔<br>Eel	975											10 slices = 50g	M058		8
白鱔<br>Japanese eel	989											10 slices = 50g	M059		8
鱸魚<br>Blace	982											1 serving = 200g	17094		8
紅衫魚/馬頭<br>Golden Thread/Horse Head	953											1 serving = 200g	M37		8
桂花魚<br>Kwai Fa Fish	996											1 serving = 200g	M38		8
生魚<br>Snake Head	984											10 slices = 50g	M068		8
鯉魚<br>Carp	981											1 serving = 200g	M061		8
塘虱<br>Catfish	980											1 serving = 200g	M27		8
石斑魚<br>Grouper	954											1 serving = 200g	17071		8
馬鮫魚<br>Mackerel	991											1 serving = 200g	M062		8
帶魚<br>Ribbon Fish	987											1 serving = 200g	M070		8
大眼魚<br>Big Eye Fish	992											1 serving = 200g	M42		8
白飯魚<br>Bak Fan Yu												1 serving = 100g	M41		8
魷魚<br>Squid	955											7 slices = 50g	19093		8
生蠔<br>Oysters	967											6 pieces = 50g	19045		8
蠔豉<br>Dried Oysters	968											10 pieces = 50g	M47		8
蝦<br>Prawns	964<br>(2pc)											2 large = 50g	19012		8
龍蝦<br>Lobsters												1 serving = 50g	19006		8
蟹<br>Crabs	969											1 serving = 100g	19033		8
蟹柳<br>Imitation Crab Sticks												1 stick = 30g	19037		8

食物種類 Type of Food	編碼 Code	過去一年的次數 How Often Within the Past Year? 從未 Never	一年幾次 A Few Times per Year	一月一次 Once per Month	一月二至三次 2 – 3 Times per Month	一星期一次 Once per Week	一星期二次 Twice per Week	一星期三至四次 3 – 4 Times per Week	一星期五至六次 5 – 6 Times per Week	每日 Every day	每次多少 How much each time?	参考份量 Reference Portion	Code	Data Entry	
		0	6	12	30	52	104	182	286	365					
帶子 Scallops	970											1 piece = 20g	19011		8
瑤柱 Dried Scallops												3 pieces = 20g	M089		8
蜆 Clams												6 pieces = 20g	19000		8
海參 (濕) Sea Cucumbers	976											1 serving = 25g	M31		8
魚丸 Fish Balls	958											5 pieces = 100g	18805		8
魚片 Fish Cakes	957 (4pc)											4 slices = 50g	18814		8
墨魚 Cuttlefish	986											7 slices = 50g	19099		8
八爪魚/墨魚/魷魚丸 Octopus/Cuttlefish/ Squid Ball												5 pieces = 100g	P1		8
鯪魚球/魚餅 Mud Carp Fish Balls	985											2 pieces = 50g	M36		8
罐頭沙丁魚 Canned Sardines	961											1 piece = 50g	17298		8
罐頭豆豉鯪魚 Fried Dace with Black Bean Sauce	974											1 piece = 50g	M48		8
吞拿魚 Tuna Fish	962 (1/3 can)											1/3 can = 50g	17026		8
咸魚 Salted Preserved Fish	977											1 slice = 5g	M29		8
海蜇 Jelly Fish	978											1 serving = 50g	M49		8
三文魚 Salmons	990											5 pieces = 100g	17054		8
炸魚 Fried Fish												1 serving = 100g	17187		8
其他 Others															
其他 Others															
其他 Others															

蛋類 Eggs

食物種類 Type of Food	編碼 Code	過去一年的次數 How Often Within the Past Year?									每次多少 How much each time?	参考份量 Reference Portion	Code	Data Entry	
		從未 Never	一年幾次 A Few Times per Year	一月一次 Once per Month	一月二至三次 2 – 3 Times per Month	一星期一次 Once per Week	一星期二次 Twice per Week	一星期三至四次 3 – 4 Times per Week	一星期五至六次 5 – 6 Times per Week	每日 Every day					
		0	6	12	30	52	104	182	286	365					
煲熟雞蛋/蒸蛋 Hard Boiled Eggs	1154											1 piece = 50g	19510		8
煎/炒雞蛋 Pan Fried Eggs	1155											1 piece = 50g	19509		8
雞蛋白 Eggs White	1152											1 piece = 35g	19522		8
雞蛋黃 Egg Yolks												1 piece = 15g	M064		8
皮蛋 Century Eggs	1157											1 piece = 50g	E2		8
咸蛋 Salted Duck Eggs	1156											1 piece = 50g	E1		8
咸蛋黃 Salted Duck Egg Yolks												1 piece = 15g	E004		8
鵪鶉蛋 Quail Eggs	1158											1 piece = 10g	19530		8
其他 Others															

奶類及飲料Dairy Products & Beverages

食物種類<br>Type of Food	編碼<br>Code	過去一年的次數<br>How Often Within the Past Year?									每次多少<br>How much each time?	參考份量<br>Reference Portion	Code	Data Entry	
		從未<br>Never	一年幾次<br>A Few Times per Year	一月一次<br>Once per Month	一月二至三次<br>2 – 3 Times per Month	一星期一次<br>Once per Week	一星期二次<br>Twice per Week	一星期三至四次<br>3 – 4 Times per Week	一星期五至六次<br>5 – 6 Times per Week	每日<br>Every day					
		0	6	12	30	52	104	182	286	365					
全脂牛奶<br>Whole Milk	76											1 cup = 250ml	1		11
脫脂奶<br>Skimmed Milk	79											1 cup = 250ml	132		11
高鈣低脂奶<br>Hi Calcium Milk												1 cup = 250ml	D020		11
朱古力奶<br>Chocolate Milk	78											1 cup = 250ml	20		11
全脂奶粉<br>Whole Milk Powder	82											1 tbsp = 7g	66		8
脫脂奶粉<br>Skim Milk Powder	83<br>(1 Tbp)											1 tbsp = 7g	203		8
高鈣脫脂奶粉<br>High CalciumSkim Milk Powder	136											1 tbsp = 7g	D013		8
煉奶<br>Condensed Milk	80<br>(1 Tbp)											1 tbsp = 20g	11		8
花奶<br>Evaporated Milk	81<br>(Tbp)											1 tbsp = 15g	15		8
芝士<br>Cheese	95											1 slice = 20g	1072		8
全脂酸乳酪<br>Whole Fat Yogurt	111											1 cup = 150g	2095		8
低脂酸乳酪<br>Low Fat Yogurt	112											1 cup = 150g	2015		8
雪糕<br>Ice Cream	99<br>(c)											1 cup = 134ml	2004		11
乳酪雪糕<br>Frozen Yogurt												1 cup = 144g	2064		8
雪糕甜筒<br>Ice Cream Cone	99<br>(cone)											1 cone = 70g	2093		8
雪條<br>Popsicles												1 piece = 88g	90722		8
奶昔<br>Milk Shake	2117											1 small cup = 300ml	2024		11
沙律醬<br>Mayonnaise	2456											1 tbsp = 15g	8021		8
朱古力粉<br>Chocolate Power Drink	2267											1 tsp = 8g	14		8
好立克<br>Horlick	2268											1 scoop = 7g	B16		8
阿華田<br>Ovaltine	2269											1 scoop = 7g	B15		8
加營素<br>Ensure	90											1 scoop = 9 g	B24		8
倍力加<br>Enercal Plus	89											1 scoop = 18 g	B25		8

食物種類<br>Type of Food	編碼<br>Code	過去一年的次數<br>How Often Within the Past Year?<br>從未<br>Never<br>0	一年幾次<br>A Few Times per Year<br>6	一月一次<br>Once per Month<br>12	一月二至三次<br>2 – 3 Times per Month<br>30	一星期一次<br>Once per Week<br>52	一星期二次<br>Twice per Week<br>104	一星期三至四次<br>3 – 4 Times per Week<br>182	一星期五至六次<br>5 – 6 Times per Week<br>286	每日<br>Every day<br>365	每次多少<br>How much each time?	參考份量<br>Reference Portion	Code	Data Entry	
可口可樂<br>Coca Cola	2251<br>(can)											1 can =<br>330ml	20147		11
其他汽水<br>Diet coke<br>Fanta Orange<br>Sprite	(can)<br>2252<br>2254<br>2253											1 can =<br>330ml	20150<br>20153<br>20163		11
益力多 (標準)<br>Yakult (Standard)	2260											1 bottle =<br>100ml	B1		11
益力多 (低糖)<br>Yakult (Low sugar)												1 bottle =<br>100ml	B059		11
維他奶 (標準)<br>Vitasoy (Standard)	2261<br>(s)											1 pack =<br>250ml	B10		11
維他奶 (低糖)<br>Vitasoy (Low sugar)												1 pack =<br>250ml	B050		11
新鮮果汁<br>Fresh Fruit Juices	2298											1 cup =<br>250ml	3090		11
罐頭盒裝果汁<br>Canned/Bottled Juices	2301											1 pack =<br>250ml	20070		11
豆漿 (標準)<br>Soy Milk (Standard)	2292											1 cup =<br>250ml	20694		11
豆漿 (低糖)<br>Soy Milk (Less sugar)												1 cup =<br>250ml	20693		11
豆漿 (無糖)<br>Soy Milk (No sugar)												1 cup =<br>250ml	20033		11
紙包/樽裝甜飲品<br>Sweetened Carton drinks/Bottled drinks	3016											1 pack =<br>250ml<br>1 bottle =<br>500ml	B5		11
中國茶 (無糖)<br>Chinese Tea	2282											1 cup =<br>250ml	B14		11
綠茶 (無糖)<br>Green Tea	2272											1 cup =<br>250ml	B17		11
西茶 (無糖)<br>English Tea	2306											1 cup =<br>250ml	20132		11
奶茶 (有奶有糖)<br>Milk Tea w/milk & sugar												1 cup =<br>250ml	B032		11
咖啡(有奶有糖)<br>Coffee w/milk & sugar	2273											1 cup =<br>250ml	B13		11
甜酒<br>Wine	2276											1 cup =<br>250ml	22507		11
烈酒<br>Spirits	2303											1 cup =<br>250ml	22661		11
低度啤酒<br>Light Beer	2275											1 cup =<br>250ml	22512		11
啤酒<br>Beer	2274											1 cup =<br>250ml	B034		11
孖蒸<br>Chinese wine	2277											1 cup =<br>250ml	22600		11

食物種類<br>**Type of Food**	編碼<br>**Code**	過去一年的次數<br>**How Often Within the Past Year?**									每次多少<br>**How much each time?**	参考份量<br>**Reference Portion**	**Code**	**Data Entry**	
		從未<br>**Never**	一年幾次<br>**A Few Times per Year**	一月一次<br>**Once per Month**	一月二至三次<br>**2 – 3 Times per Month**	一星期一次<br>**Once per Week**	一星期二次<br>**Twice per Week**	一星期三至四次<br>**3 – 4 Times per Week**	一星期五至六次<br>**5 – 6 Times per Week**	每日<br>**Every day**					
		0	6	12	30	52	104	182	286	365					
其他酒精飲品<br>Others (wine- all tables)												1 cup = 250ml	22577		11
礦泉水<br>Mineral Water	2305											1 cup = 250ml	440385		11
清水<br>Plain Water	W1											1 cup = 250ml	20041		11
菊花/清涼茶 (甜)<br>Chrysanthemum tea (sweetened)												1 cup = 250ml	B4		11
其他<br>Others															

小食類 Snacks

食物種類<br>Type of Food	編碼<br>Code	過去一年的次數<br>How Often Within the Past Year?									每次多少<br>How much each time?	参考份量<br>Reference Portion	Code	Data Entry	
		從未<br>Never	一年幾次<br>A Few Times per Year	一月一次<br>Once per Month	一月二至三次<br>2 – 3 Times per Month	一星期一次<br>Once per Week	一星期二次<br>Twice per Week	一星期三至四次<br>3 – 4 Times per Week	一星期五至六次<br>5 – 6 Times per Week	每日<br>Every day					
		0	6	12	30	52	104	182	286	365					
雲吞<br>Wonton	3066											1 piece = 20g	P7		8
叉燒包<br>BBQ Pork Bun	2004 (l)											1 piece = 75g	P213		8
蓮蓉包/奶黃包<br>Sweet Bun w/ Lotus Seed Paste/Egg Yolk Filling	2009											1 piece = 40g	P162		8
燒賣/蝦餃/粉果<br>Steamed Dim Sum	3060											1 piece = 20g	P70		8
春卷/咸水角<br>Deep Fried Dumplings	3064											1 piece = 35g	P43		8
腸粉<br>Steamed Cheung Fan (Steamed Rice Rolls)	3062											1 piece = 60g	P72		8
籮蔔糕<br>Chinese Turnip Cake	2018											1 piece = 40g	P12		8
鳳爪<br>Chicken Paw	2010											1 plate = 25g	M091		8
糯米雞<br>Sticky rice dumpling												1 piece = 275g	P214		8
油炸鬼<br>“Yau-Char-Kwai” (Deep Fried Dough)	2020											1 piece = 70g	P37		8
意大利薄餅<br>Pizza	2134 (reg)											1 piece = 120g	56483		8
熱狗<br>Hot dog												1 piece = 100g	66004		8
漢堡包<br>Hamburger	2101											1 piece = 100g	69008		8
魚柳包<br>Fish Burger	2109											1 piece = 140g	69013		8
麥樂雞<br>Chicken Nuggets	2108											1 piece = 20g	15174		8
薯餅<br>Hash Brown	2121											1 piece = 50g	6155		8
豬肉批<br>Pork Pie	2042											1 piece = 100g	56232		8
蘋果批<br>Apple Pie	2112											1 piece = 77g	48136		8
牛肉乾<br>Glazed Beef Jerky	564											3 pieces = 100g	M065		8
豬肉乾<br>Glazed Pork Jerky	579 (3pc)											3 pieces = 100g	M066		8
牛肉鬆<br>Beef Floss	563											1 pack = 25g	M33		8
豬肉鬆<br>Pork Floss	579 (1pack M)											1 pack = 25g	M18		8
食物種類	編碼	過去一年的次數									每次多少	参考份量	Code	Data Entry	

Type of Food	Code	How Often Within the Past Year?									How much each time?	Reference Portion			
		從未<br>Never	一年幾次<br>A Few Times per Year	一月一次<br>Once per Month	一月二至三次<br>2 – 3 Times per Month	一星期一次<br>Once per Week	一星期二次<br>Twice per Week	一星期三至四次<br>3 – 4 Times per Week	一星期五至六次<br>5 – 6 Times per Week	每日<br>Every day					
		0	6	12	30	52	104	182	286	365					
紫菜<br>Seaweed, dried												1 serving = 1g	V11		8
魷魚絲<br>Dried Squid Strings	956											1 pack = 20g	19073		8
糖水<br>Chinese Sweet Soup Desserts	2016											1 bowl = 200g	L12		8
豆腐花<br>Tofu Fa	1555											1 bowl = 200g	L1		8
咸餅乾<br>Saltines/Cream Crackers	287											3 pieces = 18g	71288		8
甜餅乾<br>Semi-sweet Biscuits	292<br>2 pc (s)											2 pieces = 20g	90164		8
朱古力餅乾<br>Chocolate Coated Biscuits	295											3 pieces = 40g	47031		8
合桃酥<br>Walnut Short Cakes	2040											1 piece = 150g	P215		8
牛奶布甸<br>Milk Pudding	120											1 cup = 120g	2609		8
蛋撻<br>Egg Tart	302<br>(l)											1 piece = 60g	P84		8
炸薯條<br>French Fries	2110<br>1 pack<br>(m)											1 med pack = 100g	5462		8
炸薯片<br>Potato Chips	2467<br>1 pack<br>(m)											1 small pack = 35g	44006		8
軟蛋糕<br>Spongy Cake	301<br>1 roll<br>(garden)											1 piece = 70g	46001		8
牛油蛋糕/西餅<br>Pound Cake	300											1 slice = 80g	46015		8
雞蛋仔/格仔餅<br>Chinese egg puff												1 piece = 120g	P23		8
朱古力<br>Milk Chocolate	2461											1 piece = 8g	23016		8
糖果<br>Candies	3017											1 piece = 5g	90671		8
蜜糖<br>Honey	2452											1 tbsp = 20g	25001		8
果醬<br>Jam	2453<br>(Tbp)											1 tbsp = 20g	23054		8
花生醬<br>Peanut Butter	2454<br>(Tbp)											1 tbsp = 15g	4627		8
糖漿<br>Corn Syrup	2457											1 tbsp = 20g	25000		8
糖<br>Sugar	2451											1 tbsp = 10g	25006		8
其他漢堡包<br>Other Burgers															8
其他<br>Others															

湯水類 Soups

食物種類<br>Type of Food	編碼<br>Code	過去一年的次數 How Often Within the Past Year?<br>從未<br>Never	一年幾次<br>A Few Times per Year	一月一次<br>Once per Month	一月二至三次<br>2 – 3 Times per Month	一星期一次<br>Once per Week	一星期二次<br>Twice per Week	一星期三至四次<br>3 – 4 Times per Week	一星期五至六次<br>5 – 6 Times per Week	每日<br>Every day	每次多少<br>How much each time?	参考份量<br>Reference Portion	Code	Data Entry	
		0	6	12	30	52	104	182	286	365					
老火湯<br>Chinese soup												1 bowl = 200ml	P85		11
滾菜湯<br>Vegetable Soup												1 bowl = 200ml	50709		11
羅宋湯<br>Borscht soup												1 bowl = 200ml	50014		11
粟米湯<br>Cream style corn												1 bowl = 200ml	P82		11
忌廉雞湯<br>Cream of Chicken Soup	J1											1 bowl = 200ml	50018		11
其他<br>Others												1 bowl = 200ml			11
												1 bowl = 200ml			11
												1 bowl = 200ml			11
												1 bowl = 200ml			11
												1 bowl = 200ml			11
												1 bowl = 200ml			11

調味料和油 Condiments and Oils

每天煮食油的用量/**Daily Cooking Oil Usage:**

	Home (including homemade food)	No. of dishes + types of usual dishes	Cooking method	Outside	No. of dishes + types of usual dishes	Cooking method
B	/7			/7		
L	/7			/7		
D	/7			/7		

	早餐 **Breakfast**	午餐 **Lunch**	晚餐 **Dinner**	每天共 **Total:**	**Code**	**Data Entry** 365	
粟米油 **Corn Oil**	湯匙 Tbsp	湯匙 Tbsp	湯匙 Tbsp	湯匙 Tbsp	8009		2
植物油 **Vegetable Oil**	湯匙 Tbsp	湯匙 Tbsp	湯匙 Tbsp	湯匙 Tbsp	90965		2
花生油 **Peanut Oil**	湯匙 Tbsp	湯匙 Tbsp	湯匙 Tbsp	湯匙 Tbsp	8026		2
芥花子油 **Canola Oil**	湯匙 Tbsp	湯匙 Tbsp	湯匙 Tbsp	湯匙 Tbsp	8084		2
橄欖油 **Olive Oil**	湯匙 Tbsp	湯匙 Tbsp	湯匙 Tbsp	湯匙 Tbsp	8008		2
豬油 **Lard**	湯匙 Tbsp	湯匙 Tbsp	湯匙 Tbsp	湯匙 Tbsp	8107		2
牛油 **Butter**	湯匙 Tbsp	湯匙 Tbsp	湯匙 Tbsp	湯匙 Tbsp	8000		2
植物牛油 **Margarine**	湯匙 Tbsp	湯匙 Tbsp	湯匙 Tbsp	湯匙 Tbsp	8061		2
其他	湯匙 Tbsp	湯匙 Tbsp	湯匙 Tbsp	湯匙 Tbsp			2

煮食鹽的用量 (鹽, 豉油, 蠔油):	Cooking Salt Usage: (Salt, Soy sauce, Oyster sauce)	Code	Data Entry 365	
❒ 淡味(2) ❒ 普通(4) ❒ 咸(6)	❒ light ❒ medium ❒ salty	53266		1

香港中文大學
矯形外科及創傷學系

關於維他命 D 與日光照射之知識及態度問卷調查

Knowledge of Vitamin D and Perceptions and Attitudes toward Sunlight Survey

Name
ID Number
Date of Birth
SCN
VDI

^Staff Initials: ________________

FFQ Completed by: self-completed

on DD / MM / YYYY

Data Entry by(^staff initials): ______________

on DD / MM / YYYY

^Initials should be written as the following format, "CTMD" for Chan Tai Man David.

1. 你的工作主要是在室內還是室外？

   a. 室內 (0)
   b. 室外 (1)

2. 在過去一年的工作日，從上午6:30至下午7:00，請問你平均有多少時間在室內?

   a. 少於一小時 (6)
   b. 一至兩小時 (5)
   c. 兩至四小時 (4)
   d. 四至六小時 (3)
   e. 六至十小時 (2)
   f. 十小時或以上 (1)

3. 在過去一年的假日，從上午6:30至下午7:00，請問你平均有多少時間在室內?

   a. 少於一小時 (6)
   b. 一至兩小時 (5)
   c. 兩至四小時 (4)
   d. 四至六小時 (3)
   e. 六至十小時 (2)
   f. 十小時或以上 (1)

4. 請問你喜歡戶外活動嗎?

   a. 是 (1)
   b. 否 (0)

5. 請問你進行戶外活動的頻密程度?

   a. 一星期兩次或以上 (6)
   b. 一星期一次 (5)
   c. 一個月兩至三次 (4)
   d. 一個月一次 (3)
   e. 一個月少於一次 (2)
   f. 從不 (1)

6. 在過去一年的工作日, 從上午6:30至下午7:00, 請問你平均有多少時間在戶外?

   a. 少於一小時 (1)
   b. 一至兩小時 (2)
   c. 兩至四小時 (3)
   d. 四至六小時 (4)
   e. 六至十小時 (5)
   f. 十小時或以上 (6)

7. 在過去一年的假日, 從上午6:30至下午7:00, 請問你平均有多少時間在戶外?

   a. 少於一小時 (1)
   b. 一至兩小時 (2)
   c. 兩至四小時 (3)
   d. 四至六小時 (4)
   e. 六至十小時 (5)
   f. 十小時或以上 (6)

8. 請問你喜歡在陽光下活動嗎?

   a. 是 (1)
   b. 否 (0)

9. 請問你會否經常用傘遮擋陽光?

   a. 是 (0)
   b. 否 (1)

10. 在過去一星期, 請問你有多少時間受陽光照射?

a. 少於十五分鐘 (1)
b. 十五至六十分鐘 (2)
c. 一至兩小時 (3)
d. 兩至四小時 (4)
e. 四至六小時 (5)
f. 六小時或以上 (6)

11. 你認為你有接觸足夠陽光嗎?

a. 是 (1)
b. 否 (0)

12. 請問你有否聽過維他命D?

a. 有 (1) (請繼續回答問卷)
b. 否 (0) (你已完成問卷, 不用回答以下問題)

12.1. 你知道維他命D有何作用嗎?

a. 知道 請註明:______________________________
b. 不知道 (0)

13. 你知道維他命D的來源嗎?

a. 知道 請註明: ______________________________
b. 不知道 (0)

14. 維他命D對骨骼是否有良好的影響?

a. 是 (1) (請繼續回答問卷)
b. 否 (0) (請繼續回答問卷)
c. 不知道 (0) (請跳到第15題)

14.1. 請問你從哪裏得知以上資訊?

a. 媒體 (1)
b. 書本 (2)
c. 醫生 (3)

d. 家人及朋友 (4)
e. 其他 請註明: ______________________________

15. 你知道陽光能提供維他命D嗎?

a. 知道 (1) (請繼續回答問卷)
b. 不知道 (0) (你已完成問卷, 不用回答以下問題)

15.1. 你認為要接觸多久的陽光才能吸收足夠的維他命D?

a. 請註明: ______________________________
b. 不知道 (0)

15.2. 請問你從哪裏得知以上資訊?

a. 媒體 (1)
b. 書本 (2)
c. 醫生 (3)
d. 家人及朋友 (4)
e. 其他 請註明: ______________________________
